# Supplementary material for: Suppression of distracting inputs by visual-spatial cues is driven by anticipatory alpha activity
Source: PLoS Biol. 2023 Mar 8;21(3):e3002014. doi: 10.1371/journal.pbio.3002014 (PMC10027229; doi:10.1371/journal.pbio.3002014)
Supplement: S1 Appendix — Section A. Mean performance for Experiments 1 and 2. Section B. Decoding for distractor and target. Section C. The analysis pipeline for CTF analysis and alpha MI. Section D. Colour space in the present study. (PDF) [file pbio.3002014.s001.pdf]

S1 Appendix, Section A:. Mean performance for Experiment 1, and 2

As shown in Figure A, a two-way repeated ANOVA with sessions (valid, invalid) and distractor presence (present, absent) as factors was conducted on the mean RT data in the behavioral control experiment. All subsequent multiple comparisons were Bonferroni corrected in current study. The significant interaction between sessions and distractor presence was found ($F_{1, 20} = 4.883$, $p = 0.039$). It suggested that distractor capture effect was influenced by cue validity, which is consistent with previous studies [1]. Post-hoc analysis showed that the attentional capture by the distractor was significantly decreased in valid session, with respect to invalid session ($t_{20} = 2.454$, $p = 0.031$, two-tailed, Cohen's $d = 0.590$). Pairwise comparisons revealed that the distractor capture effect was significant in invalid-cue sessions ($t_{20} = 5.454$, $p < 0.001$, two-tailed, Cohen's $d = 1.190$), whereas the effect was not significant in valid-cue sessions ($t_{20} = 1.999$, $p = 0.059$, two-tailed, Cohen's $d = 0.436$). This result showed that a distractor could be proactively inhibited when a spatial cue was presented that indicated the location of the distractor.

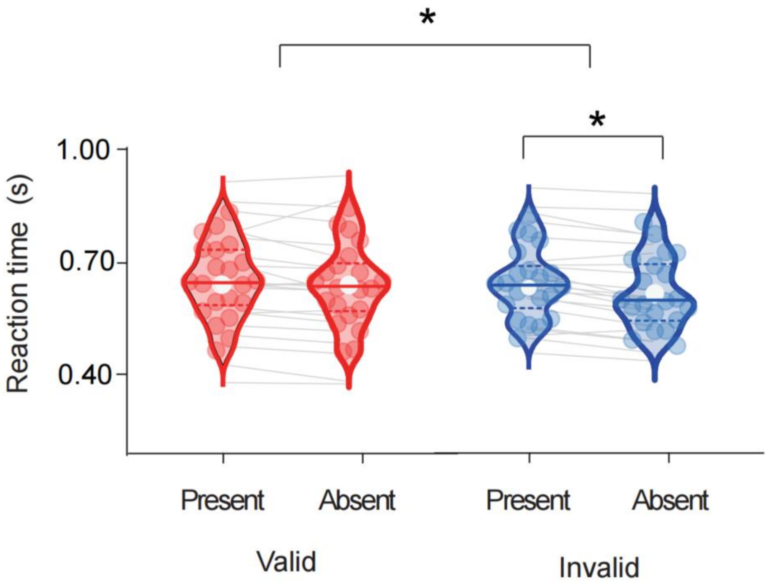

Figure A. Mean reaction times from the behavioral control experiment * $p < 0.05$ (see S7 Data for raw values).

For Experiment 1, the mean behavior outcomes for the two sessions in Experiment 1 is shown in Figure B. No significant distractor cueing effects (valid − invalid) were found for mean ACC, mean RT and mean ES (ps > 0.231, $BF_{10} < 0.258$). Our behavioral result in Experiment 1 was consistent with previous studies [2-4] that they found there was no difference between validly and invalidly cued condition.

For Experiment 2, behavioral results (Figure B) showed a main effect of predictive validity on mean ES ($F_{2, 50} = 6.449$, $p = 0.003$, $\eta^2 = 0.205$). Planned pairwise comparisons for ES again showed a prominent cueing effect on ES in high predictive validity trials (High minus Null: $t_{25} = 2.501$, $p = 0.019$, two-tailed, Cohen's $d = 0.492$) and low predictive validity trials (Low minus Null: $t_{25} = 3.467$, $p = 0.001$, two-tailed, Cohen's $d = 0.680$). However, no significant difference between High-

and Low-predictive validity trails was observed for the ES (t_{25} = 0.966, p = 0.342, two-tailed, Cohen's d = 0.190).

A two-way mixed-effect ANOVA with distractor predictability (Valid, Invalid; within-subjects) and target predictability (Experiment 1, Experiment 2; between-subjects) as factors was conducted on reaction time. The results revealed a significant distractor predictability by target predictability interaction ($F_{1,\ 55}$ = 4.581, p = 0.023). We suggest that general behavioral changes might depend on predictability of both target and distractor, and their relationship, which was consistent with previous studies [4,5].

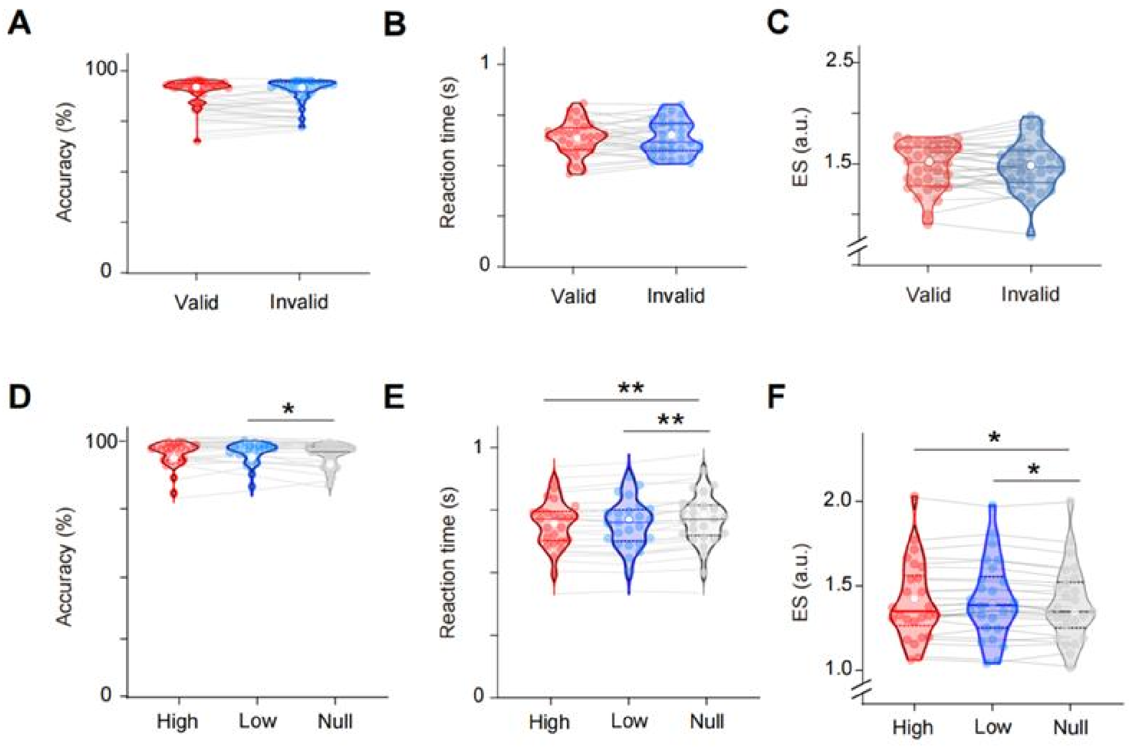

Fig B. Mean performance for Experiment 1 and 2. The mean accuracy (**A**), reaction times (**B**) and efficiency scores (**C**) in valid- and invalid-cue session for Experiment 1. The mean accuracy (**D**), reaction times (**E**) and efficiency scores (**F**) in high- , low-

and null predictive validity for Experiment 2. The solid and dotted lines indicate medians and quartiles, respectively. *p < 0.05 (see S8 Data for raw values).

S1 Appendix, Section B:. Decoding for distractor and target

As shown in Figure C, AUC (area under ROC curve) scores of distractor decoding were significantly different from the chance level in both valid-cue (142–320 ms, permutation test: $p < 0.001$) and invalid-cue sessions (154–322 ms, permutation test: $p < 0.001$). AUC scores for target location also showed a significant difference from chance level in the two sessions (valid: 236–400 ms, cluster-based permutation test: $p < 0.001$; invalid: 232–400 ms, cluster-based permutation test: $p < 0.001$). The decoding performance for the target was significantly better in valid-cue sessions from 268 to 400 ms (Figure C, right panel, cluster-based permutation test: $p < 0.01$).

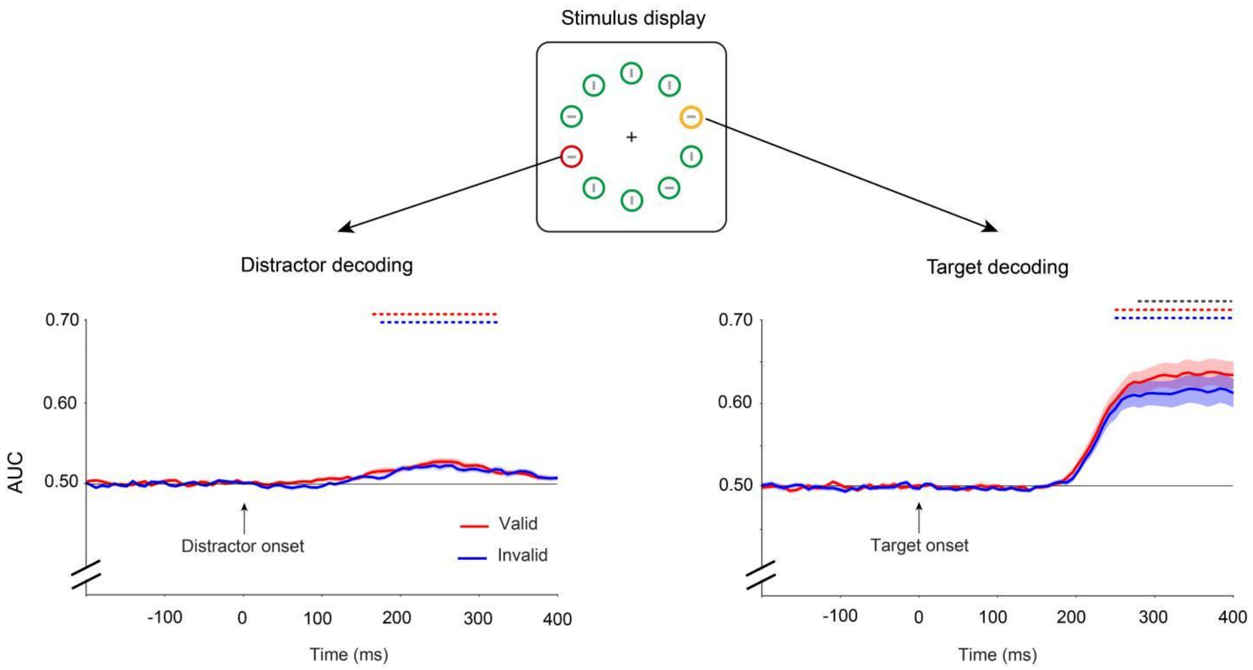

Fig C. Multivariate decoding during the stimulus period from Experiment 1. AUC scores of distractor (left panel) and target (right panel) spatial location decoding across time in valid (red) and invalid (blue) cue sessions. Shades of light color along with the dark color lines represent error bars (±1 SEM). The red and blue dashed lines at the top indicate clusters where sessions differed significantly from chance after cluster correction ($p<0.05$), and significant differences between sessions are marked by the black dashed line ($p<0.05$) (see S9 Data for raw values).

When the spatial cues were predictive, our results showed a better target decoding performance relative to invalid cues (Figure C). These findings may provide further support for the idea that cueing distractors can improve attention allocation to the target [6]. However, poststimulus distractor decoding performance did not differ. One possible reason may be the low signal-to-noise ratio of distractor-related activity. Previous studies [7] have shown that the strength of alpha lateralization for target selection was larger than that for distractor suppression. In practice, effects related to target processing are often considerably larger than effects related to distractor suppression (see rule 9 in [8]). The target decoding performance far outweighs the max value of the distractor (see Figure C) based on the same EEG datasets. Similar to winner-takes-all, this suggests that the location and feature of the target will take almost all attentional resources as long as the target appears, which results in an overwhelming target-related activity and easily drowned distractor-related activity. In this sense, the combination of a lateral distractor and a midline target, as shown in Figure 3B, enables the isolation of lateralized activity in response to the distractor. This allowed

us to examine distractor-related processes and avoid mixed target-related activity.

S1 Appendix, Section C:. The analysis pipeline for CTF analysis and alpha MI

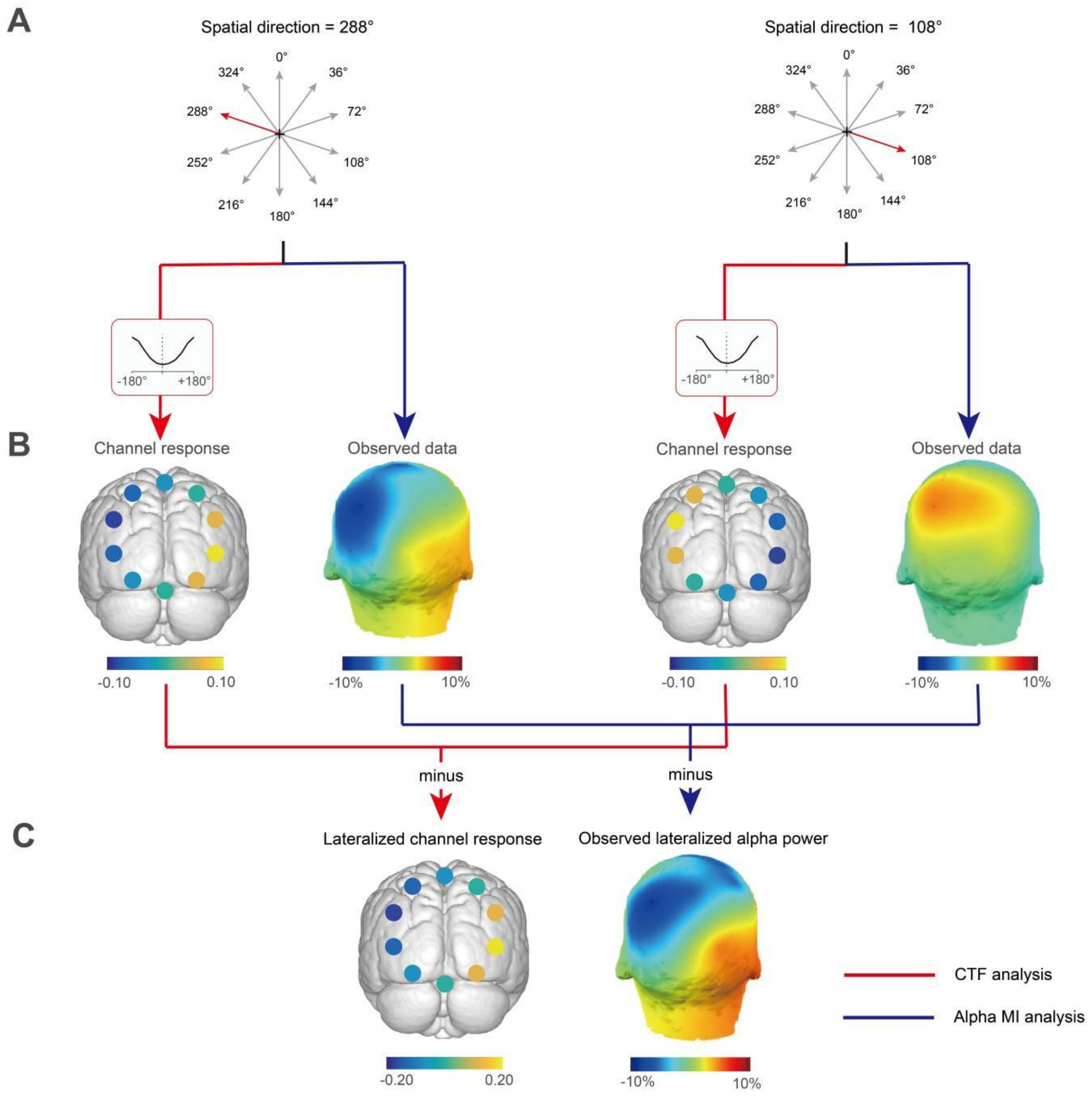

Fig D. The analysis pipeline for CTF analysis (red line) and alpha MI (blue line). (**A)** The example shown here is for a pair of spatial directions pointed at 288° and 108°. (**B)** To show spatial gradient effects related to distractor cues, the spatial change in

alpha power was rendered by mapping the channel response curve to ten ideal spatial channels. The scalp topographies show the distribution of alpha power over the posterior cortex. (**C**) The resulting lateralized channel response and observed lateralized alpha power reflect a similar spatial pattern (see S10 Data for raw values).

S1 Appendix, Section D: Colour space in the present study

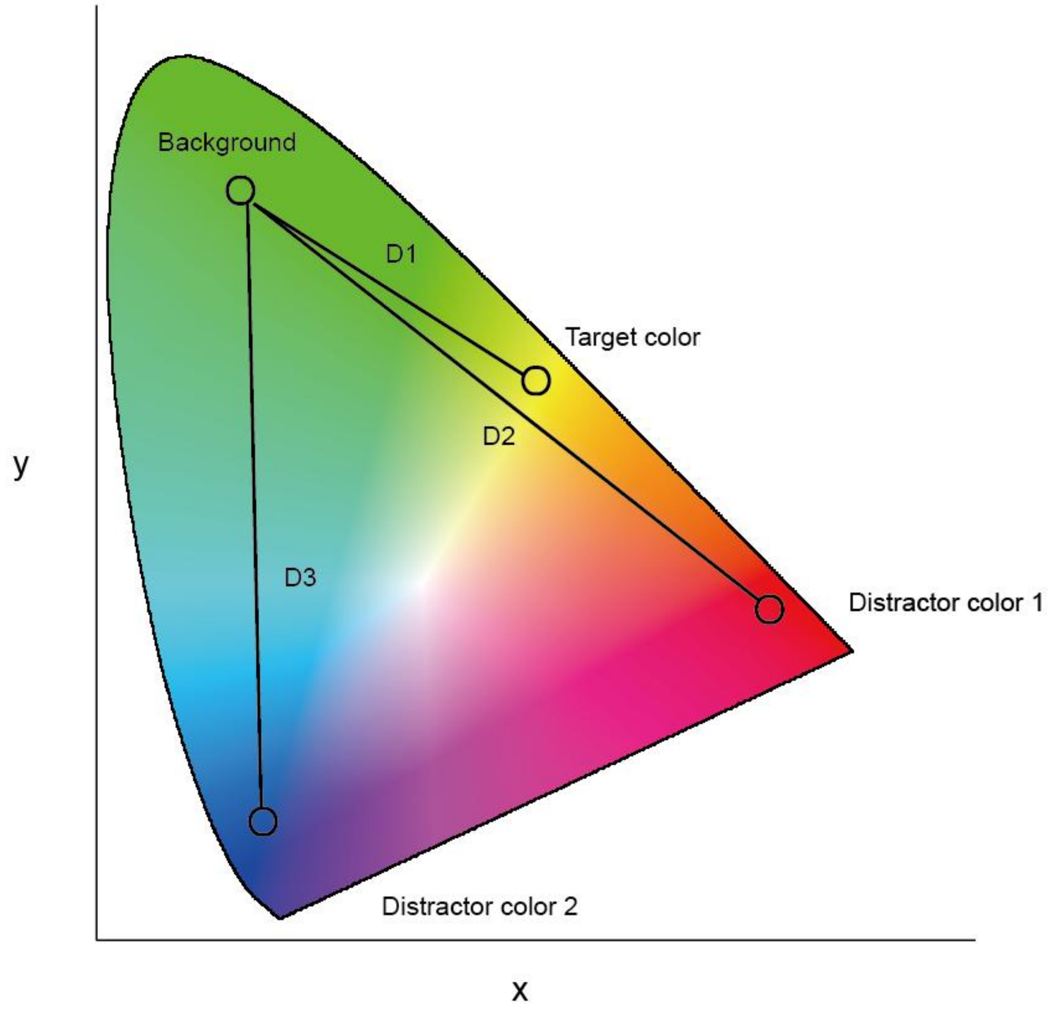

Fig E. Colour space in the present study. D1: the chromaticity space color distance between the yellow target circle and green circles. D2: the chromaticity space colour distance between the red distractor and green circles in Experiment 1, 2 and 3. D3: the chromaticity space colour distance between the blue distractor and green circles in the behavioral control experiment (see S11 Data for raw values).

Reference

1. Chang, S., Cunningham, C. A., & Egeth, H. E. (2018). The power of negative thinking: Paradoxical but effective ignoring of salient-but-irrelevant stimuli with a spatial cue. Visual Cognition, 27: 1–15.

2. Heuer, A., & Schubö, A. (2020). Cueing distraction: electrophysiological evidence for anticipatory active suppression of distractor location. Psychological Research, 84: 2111-2121.

3. van Moorselaar, D., Lampers, E., Cordesius, E., & Slagter, H. A. (2020). Neural mechanisms underlying expectation-dependent inhibition of distracting information. Elife, 9: e61048.

4. Wang, B., & Theeuwes, J. (2018). How to inhibit a distractor location? Statistical learning versus active, top-down suppression. Attention, Perception & Psychophysics, 80(4), 860 – 870.

5. van Moorselaar, D., Lampers, E., Cordesius, E., & Slagter, H. A. (2020). Neural mechanisms underlying expectation-dependent inhibition of distracting information. eLife, 9, e61048.

6. Arita, J. T., Carlisle, N. B., & Woodman, G. F. (2012). Templates for rejection: configuring attention to ignore task-irrelevant features. Journal of Experimental Psychology: Human Perception and Performance, 38(3), 580–584.

7. Wöstmann, M., Alavash, M., & Obleser, J. (2019). Alpha oscillations in the human brain implement distractor suppression independent of target selection. The Journal of Neuroscience, 39(49), 9797–9805.

8. Wöstmann, M., Störmer, V.S., Obleser, J., Addleman, Andersen, S.K., Gaspelin, N., Geng, J.J., Luck, S.J., Noonan, M.P., & Slagter, H.A., et al. (2022). Ten simple rules to study distractor suppression. Progress in Neurobiology, 213, 102269.
