# Supplementary material for: Suppression of distracting inputs by visual-spatial cues is driven by anticipatory alpha activity
Source: PLoS Biol. 2023 Mar 8;21(3):e3002014. doi: 10.1371/journal.pbio.3002014 (PMC10027229; doi:10.1371/journal.pbio.3002014)
Supplement: Attachment — Submitted filename: Response_letter R1.docx [file pbio.3002014.s013.docx]

Please address reply to:

YAN SONG

STATE Key Laboratory OF COGNITIVE Neuroscience and learning

Beijing normal university

BEIJING,100875

PEOPLE’S REPUBLIC OF China

Telephone: (8610) 5880-4267

Fax: (8610) 5880-6154

Email:songyan@bnu.edu.cn

PLoS Biology	Nov 15, 2022

**Ms. No.PBIOLOGY-D-22-01312_R1**

Title: Neural mechanisms underlying distractor suppression guided by visual-spatial cues

Corresponding Author: Prof. Yan Song

Authors: Chenguang Zhao, Yuanjun Kong, Dongwei Li, Jing Huang, Lujiao Kong, Xiaoli Li, Ole Jensen, Yan Song

Dear Editor and Reviewers:

We greatly appreciate the constructive comments from you and three reviewers. Since we received your comments, we have made a serious revision of our manuscript. 1) According to Reviewer #1’s constructive suggestions, we have added the corresponding analyses by testing the relevance of two electrophysiological signals for behavioral performance. 2) We have completed an additional behavioral control experiment (N = 20) and added new analyses and discussion to address John Foxe's concerns about the post hoc nature of some analysis choices. 3) We have added new analyses and discussion to address Clayton Hickey's concerns regarding interpretation of the ERP results. 4) A language editing service (American Journal Experts) has been used to correct the grammatical errors and check all details.

**Point-by-point changes**

**Reviewer #1:**

1. I have only one major point to make. In my opinion, the analyses miss an important opportunity by not testing the relevance of these two electrophysiological signals for behavioral performance. Given that the experiments were specifically designed to induce behavioral distraction effects, and given that alpha lateralization and Pd respond to the distraction cues, it would be very relevant to know if alpha and Pd are in turn related to behavior. Thus, I urge the authors to correlate alpha lateralization and Pd to behavioral performance either across single trials or across observers.

**Response:** In the original manuscript, we tested the relevance of these two electrophysiological signals (alpha lateralization and P_D_) to behavioral performance (the slope of ES) across observers and reported the following results:

1)“Behaviorally, we further investigated whether the slope of ES was correlated with the slope of CTF or the amplitude of P_D_ in Experiment 1, but no correlation was found across different sessions (ps > 0.684).”

2)“Behaviorally, we further investigated whether the slope of ES was correlated with the alpha MI or the amplitude of P_D_ in Experiment 2, but no correlation was found across different conditions (ps > 0.451).”

3)“Behaviorally, we further investigated whether the slope of ES was correlated with the alpha MI or the amplitude of P_D_ in Experiment 3, but no correlation was found (p = 0.331).”

These results are listed in the Results section under the subheading “Correlation analysis between alpha activity and distractor-elicited P_D_” in the original manuscript. In these cases, they may be ignored.

According to Reviewer #1’s constructive suggestions, at the single-trial level, we further investigated whether alpha lateralization and subsequent P_D_ predicted final behavioral performance by the same quartile analysis as reported in van Dijk’s research (2008). No significant difference was found among the quartiles (Figure L1, ps > 0.050) in Experiment 2 or Experiment 3.

**Figure L1.** Relationship between electrophysiological signals and behavioral performance at the within-subjects level. Averaged single-trial reaction time (left panel) and accuracy (right panel) were sorted according to cue-induced alpha MI for each quartile in high-predictive validity trials (A) and low-predictive validity trials (C) and Experiment 3 (E). Averaged single-trial reaction time (left panel) and accuracy (right panel) were sorted according to P_D_ for each quartile in high-predictive validity trials (B) and in low-predictive validity trials (D) and in Experiment 3 (F).

Furthermore, we investigated whether the cueing effects on ES slope were correlated with the cueing effects on alpha CTF slope in Experiment 1. A negative correlation (r = −0.430, p = 0.023; two-tailed) indicated that the larger the difference in the negative-going alpha CTF slope between valid cues and invalid cues, the larger the cueing effects on the ES slope (Figure L2). We suggest that the spatial distribution of the alpha power response for an upcoming distractor can influence the spatial proximity of the distractor.

**Figure L2.** Correlation between alpha CTF slope and efficiency scores (ES) slope. The ES difference between valid and invalid cues (cueing effects) varies as a function of the alpha CTF slope difference between valid and invalid cues. Positive values of the y-axis indicate larger ES slope with valid cues than with invalid cues. Negative values of the x-axis indicate that the negative-going alpha CTF slope was larger with valid cues than with invalid cues.

We discussed the above results in the Discussion: “In control behavior experiment and Experiment 2, we also found such spatial proximity of the distractor. The relationship between CTF slope and ES slope provides a neurological account for such behavioral phenomena that the spatial distribution of alpha power response for upcoming distractors can influence the spatial proximity of the distractor. With respect to the invalid session, the channel response relatively increased as alpha power decreased at electrodes contralateral to the cued distractor location, where the target was enhanced more easily. Thus, we observed that the cue is more effective when it predicts distractors appearing further away from the target. In contrast, the channel response relatively decreased as alpha power increased at electrodes ipsilateral to the cued distractor location, where the target was suppressed more easily. Thus, the cue is less effective when it predict distractors appearing closer to the target.” [Please see lines 17-23 of Page 21, lines 1-4 of Page 22 in the new version of the manuscript]

We have rewritten the above Results under the new subheading “Correlation analysis between behavioral performance and electrophysiological signals”:

1) For Experiment 1: “We further investigated whether the behavioral performance was correlated with the cue-induced alpha activity or distractor-elicited P_D_. For reaction time, accuracy, mean ES, and slope of ES, no correlation was found at the between-subject level (ps > 0.341). At the single-trial level, no significant difference was found among the quartiles for reaction time and accuracy (ps > 0.120). Interestingly, the cueing effects on the ES slope were correlated with the cueing effects on the alpha CTF slope. A negative correlation (r = −0.430, p = 0.023; two-tailed) indicated that the larger the negative-going alpha CTF slope with valid cues relative to invalid cues, the larger the cueing effects on ES slope (Figure 2E). We suggested that the spatial distribution of alpha power response for an upcoming distractor influenced the spatial proximity of the distractor.” [Please see lines 22-23 of Page 13, lines 1-8 of Page 14 in the new version of the manuscript]

2) For Experiment 2: “We further investigated whether behavioral performance was correlated with the cue-induced alpha MI or distractor-elicited P_D_. For reaction time, accuracy, mean ES, and slope of ES, no correlation was found at the between-subject level (ps > 0.161). At the single-trial level, no significant difference was found among the quartiles for reaction time and accuracy (ps > 0.330). ”

Minor：

2. Results Experiment 1: "The inverted encoding model (IEM) analysis was applied to reconstruct the attended distractor location from the pattern of alpha power to explore spatial selectivity. As shown in Figure 2A, this procedure produces CTFs, which reflect the spatial distribution of alpha power that is measured by scalp EEG (conceptualized into ten ideal electrodes)." I am a bit confused about this formulation. If I understand correctly, the cue informs observers about the location of the distractor, not the target. Thus, isn't it appropriate to refer to this as the unattended/inhibited distractor location?

**Response:** We are grateful to Reviewer #1 for reminding us that the statement of the formulation “attended distractor location” may be ambiguous. Your understanding of the Results in Experiment 1 was correct. It was intended that the cue should inform observers about the location of the distractor, not the target. Again, we thank Reviewer #1 for the new “unattended/inhibited distractor location” formulation. We have replaced “attended distractor location” with “inhibited distractor location” in the new version of the manuscript. [Please see line 11 of Page 9, line 6 of Page 10, line 15 of Page 11 in the new version of the manuscript]

3. Experiment 3: "the purpose of Experiment 3 was twofold: 1) further investigate the alpha power modulation of the distractor cue by using the arrow cue to rule out any graph-specific effects…". What difference does it make whether the cue looks like an arrow or a wedge? After all, both cue shapes are graphic and have a familiar meaning. Why would we expect that the results would be specific for either cue shape? Please elaborate.

**Response:** We are grateful to Reviewer #1 for reminding us of the potential confusion about the difference in cue shapes. We have added the following sentences to the purpose of Experiment 3: “Previous works have shown that varying shapes of cues might have different impacts on the allocation of spatial covert attention (Ortega-Alvarez et al., 2022; Lukashova-Sanz et al., 2021). In particular, radar might have different levels of distraction due to the referential position of the subjects with respect to the main scene (Ortega-Alvarez et al., 2022). It is unclear whether the results from Experiments 1 and 2 were specific to circular radar-like cues or more generally applicable......”. Thus, we further investigated the alpha power modulation of the distractor cue by using the arrow cue to rule out any shape-specific effects.

4. "Based on this model, alpha power increases at electrodes far away from the cued distractor and decreases at electrodes close to the cued distractor". I must say that I find this formulation (similar statements are found throughout the ms) misleading. The distractor is an event on the monitor, while electrodes are on the observer's head. I don't think it makes much sense to say that an electrode is close or far from a distractor. Moreover, I find this makes it harder to understand the significance of the finding, which is that alpha increases *contralateral* to the distractor, in other words in the hemisphere representing that distractor. I strongly suggest rephrasing these statements.

**Response:** We are grateful to Reviewer #1 for reminding us that this formulation is misleading. According to Reviewer #1’s constructive suggestion. We have rephrased these statements:

1) We have replaced “Based on this model, alpha power increases at electrodes far away from the cued distractor and decreases at electrodes close to the cued distractor" with “Based on this model, the amplitude and spatial distribution of the alpha band over the scalp has a graded change. Alpha increases were maximal over occipitoparietal electrodes contralateral to the cued distractor location and maximum alpha decreases were found ipsilaterally to the cued distractor location.” [Lines 20-23 of Page 23 in the new version of the manuscript].

2) We have replaced “As shown in Figure 2D, channel response relatively increased at electrodes far away from the cued distractor location, channel response relatively decreased at electrodes close to the cued distractor location.” with “As shown in Figure 2D, channel response relatively increased at channels contralateral to the cued distractor location, and channel response relatively decreased at channels ipsilateral to the cued distractor location.” [Lines 3-5 of Page 11 in the new version of the manuscript]

**Reviewer #2 [John J. Foxe]:**

1. The use of Efficiency Scores to represent behavioural performance strikes me as non-standard and rather post-hoc. Is there a precedent for this measure of behavior and what is the rationale? At the very least, we should be able to see both the accuracy and RT data independently in the main document. It becomes clear from Figure S1 that neither accuracy nor RT are significantly affected by the experimental manipulation. In these cases, it would also be important to fully report the statistics.

**Response:** We are grateful to Reviewer #2 for reminding us that the analytic rationale for the efficiency scores is not clear. We have added the data and statistics of the slope of accuracy and RT data independently in the main document (see Figure 1). We have also added the data and statistics of mean accuracy and RT independently in the supplementary materials (See Figure S1).

Multivariable behavioral data or potential speed/accuracy trade-offs tend to complicate interpretation. Efficiency scores would be simple if accuracy and reaction time could be integrated into a single dependent variable, which appropriately weighs the impact of speed and accuracy. Such a measure was first proposed by Townsend and Ashby et al. (1978, 1983). Since then, there have been many contributions to this measure of behavior (Akhtar and Enns, 1989; Kennett et al., 2001; Jacques et al., 2007; Langner et al., 2015; Li et al., 2016; Salamone et al., 2021; Cazzato et al., 2021). Therefore, the use of efficiency scores to represent behavioral performance is standard rather than post hoc.

Efficiency scores are a better measure of behavioral data when the number of errors is small and when there is a high correlation between RT and ACC (Statsenko et al., 2020). In the present study, the behavioral data showed low errors (< 5%), and the same tendency was observed in both RT and ACC. Please see RT and ACC across the distances of the target to the distractor location (DTD) shown in Figure 1. The response to the target showed an apparent RT disadvantage and accuracy disadvantage when distractors occurred at locations (the 1st and 2nd) close to the target. The response to the target showed an apparent RT advantage and accuracy advantage when distractors occurred at locations (the 5th and 4th) far away from the target. Accordingly, “to take multivariable behavioral data into account, we further used efficiency scores (ES) by dividing the ACC by RT (see Townsend & Ashby, 1978), which is a standard way to combine ACC and RT measures of performance. A larger ES means more efficient responses.” We have added the above sentences in lines 9-12 of Page 7 in the new version of the manuscript.

2. I found it very difficult to unpack precisely what the paradigm was and what exactly the participants were instructed to do. This really needs a much better and more detailed description. There is about half a paragraph devoted to describing the paradigm in Experiment 1 that is thoroughly opaque, and the associated figure does not make things a whole lot clearer. The order of valid and invalid cue sessions was counterbalanced across subjects, but did which one was run first have any impact on the results? How did subjects know which one they were to do? Were they informed or did they simply start and learn it implicitly? This is important because one can easily imagine a subject completing session one where the cues are fully uninformative and then taking many trials on session two (a week later) to discover that the cues now have meaning. That the paradigm figure, experimental description and the figure legend were all in different places in the document made this even more difficult to figure out.

**Response:** Thank you for reminding us that the description of the paradigm in Experiment 1 is not clear. To answer the question of whether the session order has any impact on the results, a two-way repeated ANOVA with session (valid, invalid) and order (valid first, valid second) as factors was conducted on behavioral performance. The results revealed no significant main effect of order (ps > 0.254) and no significant session-by-order interaction (ps > 0.431), suggesting that which one was run first has no impact on the results.

Before each session, participants were informed of the cue validity (valid or invalid) and its corresponding spatial probability to the target and distractor, which guided participants to indicate the orientation of the gray line inside the yellow target circle in the search array. Therefore, subjects did not need to learn what the cues meant.

According to Reviewer #2’s suggestion, we have added more detailed descriptions of the paradigms, including the paradigm figure, experimental description, and figure legend:

1) “Before each session, participants were informed of the cue validity (valid or invalid) and its corresponding spatial probability of the target and distractor (Figure 1B). Participants completed some practice trials to ensure that they understood the task requirements and learned how to make good use of cues.” [Lines 21-23 of Page 35, line 1 of Page 36 in the new version of the manuscript]

2）We have added more detailed figure legends about the target and distractor in Figure 1A. [Please see Fig. 1 in the new version of the manuscript]

3）We have added “Before each session, participants were informed of the cue validity (valid or invalid) and its corresponding spatial probability of the target and distractor in advance. ” in Figure 1B’s legend.

4) We have added “Before each session, participants were informed of the cue validity (valid or invalid) and its corresponding spatial probability of the target and distractor, which guided participants to indicate the orientation of the gray line inside the yellow target circle in the search array. Participants were encouraged to make use of the information provided by the predictive cues, which would help them not to get distracted by the salient distractor.” in the Results section. [Lines 15-20 of Page 6 in the new version of the manuscript]

3. Was the distractor actually red? If so, how can this really be considered an effective distractor? Doesn't the distractor "declare itself" by simply being a different colour? Given that it appears that participants receive all the information they need from the appearance of the coloured rings themselves, what use to them is the cue? That is, it may simply be much easier for them to wait for the appearance of the very easily dissociated colour rings before deploying attention than to expend neural resources suppressing a distractor space that will declare itself. Frankly, I am not convinced at all of the efficacy of the design, and the general lack of strong behavioural changes seems to back this view up.

**Response:**

**1) About the color of distractor**

The distractor was actually red in the original version of the manuscript. We already explained how a red distractor can be considered an effective distractor in the original manuscript: “Salience was defined in terms of the local contrast between green circles and each color circle (see Figure S5): the distance in chromaticity space between the red distractor (RGB: 255, 100, 100) and green circles (RGB: 0, 180, 0) was greater than the distance between the yellow target (RGB: 160, 160, 0) circle and green circles. A red distractor with more salience captured attention more easily than a yellow target, creating more incentive to ignore distracting sensory information.” [Please see lines 16-23 of Page 35, line 1 of Page 36 in the new version of the manuscript].

Recently, eleven experts from the distractor-related field offered ten simple rules to study distractor suppression in *Progress in Neurobiology* (Wöstmann et al., 2022). They wrote that “a red stimulus will likely be more distracting when surrounded by green versus orange stimuli.” in Rule 1 (make sure the distractor has the potency of a distractor). Several previous studies applied such a color combination (red distractor, green background, less salient yellow target) to investigate the mechanism underlying distractor inhibition (Gaspar et al., 2014; Fortier-Gauthier et al., 2013; Pomerleau et al., 2014). According to Reviewer #2’s suggestion, we have added other color distractors in an additional experiment (for details, please see the following point).

**2) About the effectiveness of distractor**

Furthermore, Reviewer #2 asked “what use to them is the cue?” We are delighted that the above question is of great interest to Reviewer #2. Several studies (Heuer & Schubö, 2020; Chang et al., 2018; Chao, 2010; Munneke et al., 2008; Noonan et al., 2016) have been conducted to directly cue the location of a distractor to exert proactive trial-by-trial adjustments of spatial suppression. Chao (2010) and Munneke et al. (2008) found that cueing distractors can facilitate target search when the distractors were not salient, which was different from our experimental design. Our behavior results in Experiment 1 were consistent with previous studies (Heuer & Schubö, 2020; Noonan et al., 2016; Wang & Theeuwes, 2018), which showed no difference between validly and invalidly cued conditions in reaction time and accuracy when the distractors were salient, which was similar to Experiment 1’s design. “It was suggested that behavioral changes (e.g., reaction time, accuracy) are only indirect indicators of distractor suppression, as they reflect the target-related outcome of all processes from stimulus presentation up until response of the task (Heuer & Schubö, 2020).” Accordingly, how the explicit distractor cue changes target-related performance is complex and remains ambiguous.

**3）About the behavioral results**

As shown in Figure L3, no significant difference between valid and invalid sessions was found in Experiment 1 when the target location was unpredictable with equal probability at each location. However, a significant cueing effect on both the mean RT and ES was found in Experiment 2 when the target location was predictable with uniform spatial probability in each location. A two-way mixed-effect ANOVA with distractor predictability (valid, invalid; within-subjects) and target predictability (unpredictable target in Experiment 1, predictable target in Experiment 2; between-subjects) as factors was conducted on reaction time. The results revealed a significant distractor predictability by target predictability interaction (F_1, 55_ = 4.581, p = 0.023). We suggest that general behavioral changes might depend on the predictability of both the target and distractor, and their relationship, which was consistent with previous studies (van Moorselaar et al., 2020; Wang & Theeuwes, 2018).

**Figure L3.** Mean performance for Experiments 1 and 2. The mean accuracy (**A**), reaction times (**B**), and efficiency scores (**C**) in valid and invalid cue sessions for Experiment 1. The mean accuracy (**D**), reaction times (**E**) and efficiency scores (**F**) in high-, low, and null-predictive validity trials for Experiment 2. *p < 0.05, **p < 0.01.

**4）Evidence supporting the effectiveness of distractor**

Previous studies have provided several direct indicators of distractor suppression as follows: 1) Eye movement. Previous research provided behavioral evidence that distractors can be suppressed proactively in trialwise analysis by showing that eye movements are less likely to be deployed to a cued distractor (van Zoest et al., 2021); 2) Distractor effect (or distractor capture). One popular but restricted approach is to omit the intended distractor in some trials by comparing the task performance of the distractor-present trials with those of the distractor-absent trials (Geyer et al., 2008; Wöstmann et al., 2022). The performance decrement for distractor-present trials is thought to quantify the distraction effect. Chang et al. (2018) recently provided behavioral evidence that an informative spatial distractor cue eliminates the distractor effect in advance. 3) EEG decoding performance. EEG decoding has proven useful for understanding the contributions of EEG signals to spatial attention. Van Moorselaar et al. (2020) recently showed that when the distractor features were predictable, they could already be decoded from the EEG activity before the search display onset. 4) The spatial proximity of the distractor. See the next Response for details.

In the current study, we provide three of the above direct indicators of distractor suppression to support the efficacy of Experiment 1’s design as follows:

1) As Reviewer #2 pointed out, “Lack of strong behavioral changes seems to back this view up”. Our behavioral results in Experiment 1 cannot provide strong behavioral changes as general behavioral changes (such as the mean RT). Therefore, “to provide general behavioral evidence and exclude the possible influence of the red color, we conducted an additional experiment. We invited 20 healthy adults to complete an additional behavioral control experiment by adding distractor-absent trials. We changed half of the blocks to blue distractors instead of red distractors to control the specific red color effect. We aimed to investigate whether a valid spatial cue can decrease the distractor effect.” The additional experiment was named the behavioral control experiment in the new version of the manuscript.

“In the behavioral control experiment, the apparatus, stimuli, and procedure were the same as in Experiment 1, except for the following: 1) We replaced the red distractor with a blue distractor in half of the blocks. It is noted that the blue distractor has the same salience as the red distractor, as the distance in chromaticity space between the red distractor and green circles was equal to the distance between the blue distractor (RGB: 45, 0, 245) circle and green circles. 2) We removed salient distractors in 20% of trials (distractor-absent trials) in both valid and invalid cue sessions. That is, in the remaining 80% of distractor-present trials, the cue display was fully predictive or not predictive of the specific location of the red or blue distractor circle in two separate sessions.” [Please see lines 18-22 of Page 36, lines 1-4 of Page 37 in the new version of the manuscript]

As shown in Figure L4, a two-way repeated ANOVA with session (valid, invalid) and distractor presence (present, absent) as factors was conducted on the mean RT data in the behavioral control experiment. All subsequent multiple comparisons were Bonferroni-corrected in the current study. A significant interaction between session and distractor presence was found (F_1, 20_ = 4.883, p = 0.039). This result suggested that the distractor capture effect was influenced by cue validity, which was consistent with previous studies (Chang et al., 2018). Post hoc analysis showed that attentional capture by the distractor was significantly decreased in valid sessions with respect to invalid sessions (t_20_ = 2.454, p = 0.031, two-tailed, Cohen’s d = 0.590). Pairwise comparisons revealed that the distractor capture effect was significant in invalid cue sessions (t_20_ = 5.454, p < 0.001, two-tailed, Cohen’s d = 1.190), whereas the effect was not significant in valid cue sessions (t_20_ = 1.999, p = 0.059, two-tailed, Cohen’s d = 0.436). This result suggested that a distractor could be proactively inhibited when a spatial cue could indicate the location of the coming distractor.

**Figure L4.** Mean reaction times from the behavioral control experiment. * p < 0.05.

2) In our study, we conducted decoding analyses with ERP waveforms across all electrodes. The decoding performance (Figure L5) showed that target decoding was higher when the distractor location was predictable (valid cue) than when it was not in invalid cue session (permutation test: p < 0.001, two-tailed). This decoding result reveals that when the distractor could be predicted, participants had a better spatial representation of the target, which supports the cueing distractor benefitting the spatial representation for target selection during the search array.

**Fig. L5** Multivariate decoding during the stimulus period from Experiment 1. AUC scores of distractor (left panel) and target (right panel) spatial location decoding across time in valid (red) and invalid (blue) cue sessions. Shades of light color along with the dark color lines represent error bars (±1 SEM). The red and blue dashed lines at the top indicate clusters where sessions differed significantly from chance after cluster correction (p < 0.05), and significant differences between sessions are marked by the black dashed line (p < 0.05).

3) “In the behavioral control experiment, we still analyzed the same slope of accuracy, RT, and ES as in Experiment 1 to examine spatial changes in behavioral outcomes. We also found a significant distractor cueing effect on the slope of ACC (t_20_ = 2.398, p = 0.026, two-tailed, Cohen’s d = 0.523, Figure L6F), RT (t_20_ = −2.451, p = 0.024, two-tailed, Cohen’s d = −0.535, Figure L6G), and ES (t_20_ = 2.778, p = 0.012, two-tailed, Cohen’s d = 0.606, Figure L6H). Similar to Experiment 1, no significant distractor cueing effects (valid − invalid) of mean ACC, RT, or ES were found (p > 0.371). These results repeated the findings in Experiment 1, suggesting that the cueing distractor explicitly truly influenced the spatial proximity of the distractor, but not in relation to the specific color.” [Please see the next Response for details].

**Fig. L6** Behavioral results for the behavioral control experiment. The slope of efficiency scores in the valid (red) and invalid (blue) cued distractor sessions for accuracy (F), reaction time (G), and efficiency scores (H). ** p < 0.01, * p < 0.05.

In sum, the above three direct indicators in Experiment 1 support the existence of proactive suppression for spatial distractor cues and extend its influence from the distractor capture to the spatial proximity of the distractor and the spatial representation of the target.

**4）Additional explanation**

In addition, Reviewer #2 provided an alternative explanation: “That is, it may simply be much easier for them to wait for the appearance of the very easily dissociated color rings before deploying attention than to expend neural resources suppressing a distractor space that will declare itself.” Thus, they did not use the cue but simply ignored it, which resulted in equivalent levels of performance in the two cueing conditions. We understand Reviewer #2's opinion. In fact, it would not be easy for subjects to wait for the appearance of the distractor. Previous studies suggested that once valid information about distractors was provided in advance, people could not immediately escape attraction by the distractor information regardless of task demands (Cunningham & Egeth, 2016; Tsal et al., 2006). An alternative explanation stems from a white-bear metaphor that distractor cueing is the visual equivalent of telling someone not to think of a white bear, and it is easy to imagine how that might lead to the opposite of the desired effect (Wegner et al., 1987). Cueing distractor information is almost paradoxical to instructing someone to prepare to ignore something. They might first try to make an effort to minimize interference from cueing distractor information (Moher & Egeth, 2012) and then actively inhibit potential distractor location. [Please see lines 20-23 of Page 25, lines 1-4 of Page 26 in the new version of the manuscript for details].

4. The authors then go on to compute a distance-to-distractor index, despite the lack of main effects upon behaviour, which strikes me as entirely post-hoc. I may be wrong about this, but there was no mention of the distance-to-distractor issue in the introduction and no hypotheses forwarded in this regard, so where did this measure come from? The factor structure of the rmANOVAs that are used to model this measure is then not explained. If there are main effects of this for both valid and invalid cues, then once again, doesn't this indicate that the main experimental manipulation hasn't worked? How many ANOVAs were conducted? Were corrections made for multiple comparisons? Again, that there is a "gradient" of interference as a function of distance to the distractor that pertains in both the valid and invalid cuing conditions points to a lack of effect of the cues. All (or most) of the effects seem to be carried by the pop-out nature of the red ring. Therefore, the authors conclusion that "Taken together, our preliminary results showed novel spatial behavioral changes, which supported the existence of proactive suppression for spatial distractor cues" does not appear to be supported by their own data. Just to belabor this point a little, looking at Figure S1, one sees that these changes in the slope of the ES are actually driven by accuracy differences that amount to 91% versus 93% in the most differential case (see the rightmost plot of Panel A), and RT differences of 10-20 ms in only a handful of the distance cases.

**Respons**e: We are grateful to Reviewer #2 for reminding us that the analytic rationale of the distance-to-distractor analysis is not clear. This index in behavior is not post hoc to understand the mechanism underlying distraction. We have added an introduction to the distance-to-distractor index and put forward the following hypotheses:

1) “The spatial proximity of the distractor plays an important role in determining how proactive suppression impacts behavior (Mounts et al., 2000; Hilimire et al., 2009). As the distance between the target and distractor increased, the accuracy of target selection improves monotonically while reaction time decrease monotonically (Caputo et al., 1998; Theeuwes et al., 2004). In recent decades, the dependency of performance on the distance between the target and distractor is calculated by the performance as a function of target-distractor distance (Koch et al., 2013; Gaspar et al., 2014; Wang & Theeuwes, 2018).” Thus, we hypothesized that cueing the distractors location explicitly would influence the spatial proximity of the distractor. [Please see lines 18-21 of Page 3, lines 1-2 of Page 4 in the new version of the manuscript].

2) “As expected, these results are consistent with previous studies (Munneke et al., 2008; Chao, 2010; Heuer & Schubo, 2020; Feldmann-Wüstefeld et al., 2021; Awh et al., 2003) and provide clear evidence for a spatial gradient of suppression surrounding salient distractors.” [Please see lines 14-16 of Page 7 in the new version of the manuscript]

Next, we are grateful to Reviewer #2 for reminding us that the factor structure of the rmANOVAs is not clear. In the original version of the manuscript, two ANOVAs were conducted, and no corrections were made for multiple comparisons. After correcting the p values for multiple comparisons with Bonferroni correction, the main effects for both valid and invalid cues were still significant.

To describe it clearly, we have reanalyzed these results as follows: “A two-way repeated ANOVA with session (valid, invalid) and DTD (1st, 2nd, 3rd, 4th and 5th) as factors was conducted on mean ESs. The significant main effect of DTD (F_4, 116_ = 12.177, p < 0.001, η^2^ = 0.925) suggested that the magnitude of ESs depended on the target-distractor distance, which is consistent with previous studies (Feldmann-Wüstefeld et al., 2021; Gaspar et al., 2014). As expected, we also found a significant session-by-DTD interaction (F_4, 116_ = 3.471, p = 0.037, η^2^ = 0.314). We suggest that cueing the distractor explicitly would influence the spatial proximity of the distractor.” In the new version of the manuscript, only one ANOVA was conducted, and there is no need for further corrections. [Please see lines 17-22 of Page 7, line 1 of Page 8 in the new version of the manuscript]

Furthermore, there is a “gradient” of interference as a function of distance to the distractor that pertains to both the valid and invalid cueing conditions, just pointing to the existence of the spatial proximity of the distractor in both cueing conditions. To the best of our knowledge, the spatial proximity of the distractor could be changed with distractor salience (Koch et al., 2013), distractor statistical learning (Wang & Theeuwes, 2018). There was no evidence that the the spatial proximity of the distractor can be eliminated by valid distractor cues. Thus, the existence of the spatial proximity did not mean a lack of effect of the cues.

As mentioned above, the additional the behavioral control experiment further confirmed the results of the previous experiment on that cueing effect on spatial behavioral changes, which supported the existence of proactive suppression for spatial distractor cues (please see above point 3 for details). In addition, it ruled out “All (or most) of the effects seem to be carried by the pop-out nature of the red ring” as a possible explanation. Therefore, our conclusion that “Taken together, our preliminary results showed novel spatial behavioral changes, which supported the existence of proactive suppression for spatial distractor cues” is justified.

In addition, as shown in Figure 1, we agree with the idea that these changes in the slope of the ES are driven by accuracy differences that amount to 91% versus 93% in the most differential case and RT differences of 10-20 ms in only a handful of the distance cases. These differences are one reason why we used efficiency scores (ES) to reach a convincing conclusion. As mentioned above, the use of efficiency scores to represent behavioral performance is standard rather than post hoc (please see Point 1 of Reviewer #2 for details).

5. The use of what are quite low-density montages is a limitation (minor) of the work. Referencing to bilateral linked mastoids is a problem for assessing lateralized activity.

**Response:** Thank you for reminding us of the potential limitation of the method. We appreciate that high-density montages would be better for the exploration of brain activity. We have added a limitation of this study to the Discussion: “However, the current study only focused on the dynamic characteristics of distractor suppression during anticipation, and the findings are limited in their low-density montages to explain EEG source localization of distractor suppression. Further high-density EEG or MEG is needed to provide a precise source map” [Please see lines 15-18 of Page 26 in the new version of the manuscript]

All electrodes, except those for monitoring eye movements, were referenced to the left mastoid during online data collection and then were off-line re-referenced to the algebraic average of the left and right mastoids (see Page 28). This approach is widely applicable to assess lateralized CTF (Foster et al., 2017a; van Moorselaar et al., 2019), lateralized ERP (Hickey et al., 2009; Gaspar et al., 2014), and lateralized alpha activity (van Moorselaar et al., 2020; Noonan et al., 2016).

6. Subtracting an averaged ERP from each trial on the assumption of a perfect stationarity of response is problematic and needs better justification. The usual approach here is to use long cue-to-target intervals so that the cue-related evoked activity is already long over before the relevant anticipatory alpha-band activity comes into play. The authors do not make clear if this was done at the individual subject basis, which is crucial, and one assumes is what was done. Nonetheless, it is absolutely necessary to make this clear.

**Response:** We are grateful to Reviewer #2 for reminding us that the analytic rationale for subtracting ERP is not clear. Here, we subtracted an individual-level ERP from each trial, which is a typical approach to ensure that the oscillatory activity was not contaminated by ERPs. Thus, it was done on an individual subject basis. We have rewritten this section as follows: “Differences in time-frequency power could be explained by event-related differences in ongoing oscillatory power (i.e., “induced” activity) or ERP exhibiting strong power in (low-) frequency components (“evoked” activity; Yeung et al., 2007; van Driel et al., 2017). To evaluate this issue, we separated nonphase-locked from phase-locked power. We subtracted the trial-average activity in the time domain from the EEG activity of every single trial, thus effectively removing any phase-locked component, so we could be certain that the resulting oscillatory activity was nonphase-locked, which was not contaminated by ERPs. This method was also widely used in recent EEG studies (Cohen & Donner, 2013; Beatty et al.,2021)”. [Please see line 4-11 of Page 31 in the new version of the manuscript]

7. The title should make it clear that the work is in the visual modality.

**Response:** Thank you for this constructive suggestion. We have revised the title to “Neural mechanisms underlying distractor suppression guided by visual-spatial cues”.

8. It is a major weakness that there is no eye-tracking and that only EOG channels have been used to correct for such movements. This is not adequate in a study of spatial attention where fixation is crucial. It is not even discussed.

**Response:** We are grateful to Reviewer #2 for reminding us of this potential limitation. To control for eye blinking or closing during the presentation of a stimulus, we first rejected all trials with signals exceeding ± 100 μV at all electrodes from −200 to 400 ms in the cue- or distractor-locked epochs. To further control for horizontal eye movements, we rejected all segments with signals exceeding ± 50 μV at HEOG electrodes from −200 to 400 ms before ERP averaging. Then, we corrected vertical eye movements using ICA analyses (without HEOG correction).

To assess whether any systematic horizontal EOG activity was present in the remaining data, we computed averaged HEOG waveforms for left and right-inhibited distractor trials. Residual activity was less than 2 μV in Experiment 1, which means that the residual eye movements were less than ± 0.3° (McDonald et al., 1999). Experiments 2 and 3 consistently demonstrated that the residual activities of HEOG were also less than 2 μV. Thus, the small horizontal eye movements in adults suggest that participants fix their eyes on the center of the screen.

On the other hand, the cue or search arrays were visible for as short as 200 ms, which might also reduce horizontal eye movements. However, we admired that extra eye-tracking must be much better than only EOG channel recording to keep spatial attention at fixation. “A previous study showed that distractors can be suppressed proactively by showing that eye movements are less likely to be deployed to a cued distractor (van Zoest et al., 2021). In addition, eye movement are linked to alpha modulation (van Zoest et al., 2021; Liu et al., 2022) and P_D_ amplitude (Weaver et al., 2017) during covert spatial attention. There was no eye-tracking recording in the current study, and only EOG channels were used to measure such movements indirectly. In the future, the combination of eye movement and EEG methods will allow us to investigate both spatial and temporal aspects of distractor inhibition simultaneously”. We have added the corresponding discussion to the manuscript. [Please see lines 18-22 of Page 26 in the new version of the manuscript]

9. "Similar to black and white" in the methods section - what does this mean? If the authors are aiming to denote the idea of a binary decision, this is not a good way to do it.

**Response:** Thank you for pointing out this problem. We used “Similar to black and white” in the original manuscript to denote the idea of a binary decision. We rewrote this sentence “It is likely that within these tasks, spatial distractor cues might indirectly provide potential spatial information about a target”. [Please see lines 18-19 of Page 34 in the new version of the manuscript]

10. There is no justification or rationale provided for applying the Inverted encoding approach.

**Response:** Thank you for pointing out this problem. We applied the inverted encoding approach for the following reasons: “Such alpha lateralization reflects the distractor-related suppression by spatial attention in line with the gating by the distractor inhibition hypothesis (Jensen et al., 2010). While the hemispheric lateralization give an indication of spatial specificity, the lateralized activity alone does not necessarily reflect a precise spatial representation (Feldmann-Wüstefeld et al., 2021). An influential line of research further focuses on the precise spatial selectivity reflected by the alpha activity by applying encoding models (EMs) which track the temporal and spatial dynamics of spatial attention (Foster et al., 2017a,b; Samaha et al., 2016; Popov et al., 2019)”. We have added the above justification and rationale to the Introduction. [Please see lines 19-23 of Page 4, lines 1-2 of Page 5 in the new version of the manuscript]

11. The Pd results (see Figure 3A) are just not convincing. The arrow denoting the latency of the Pd, for example, points to a timepoint where there is no obvious distinct component and no really obvious difference between the waveforms, despite the shading. In the methods, the period used to derive the Pd is not justified (i.e. the most positive difference between P7 and P8 between 220 and 320 ms), and then a difference is ascribed to just the period between 248−316 ms. The lack of a Pd difference in this timeperiod in the valid cue condition seems to be driven by a more negative-going waveform in the ipsilateral trace in the 220-250 ms period. What are we to make of this difference? The correlations plots are wholly unconvincing, and I couldn't find any correction for multiple testing. That the MI measure showed absolutely no correlation is not surprising and further undermines the marginal finding in the case of the valid cues for the CTF slope.

**Response:** We are grateful to Reviewer #2 for reminding us of the omission of important information in Figure 3. All latencies were identified using standard cluster-based nonparametric tests (Maris and Oostenveld, 2007). First, we estimated the t statistic across participants for a contrast of interest at each time point, then defined observed clusters of consecutive above-threshold time points, and calculated the cluster mass (by summing all t values in an above-threshold cluster). Next, we randomly shuffle condition labels within participants (sign-flip for contrasts against zero) and extracted the largest cluster mass produced by chance. This permutation step is performed 10,000 times to estimate the null distribution. The probability of the observed group-level cluster against chance is then derived as the rank order of the observed cluster relative to the null distribution. We have added some details to the manuscript as follows:

“Figure L7 shows difference waveforms (contralateral minus ipsilateral), revealing that a significant positive difference (248−316 ms; cluster-based permutation test: p < 0.050) in the invalid cue sessions and a significant cueing effect on difference waveforms was apparent at P7/8 electrodes (258−302 ms; cluster-based permutation test: p < 0.050). The amplitude of P_D_ was significant in the invalid cue session (t_29_ = 2.228, p = 0.034, two-tailed, Cohen’s d = 0.414) but not in the valid cue session (t_29_ = −0.007, p = 0.995, two-tailed, Cohen’s d = −0.001), which resulted in a significant P_D_ difference between the two sessions (t_29_ = −2.090, p = 0.046, two-tailed, Cohen’s d = −0.388).”

**Fig. L7** ERP results during the stimulus period from Experiment 1. (C) The left panel shows the corresponding difference waves (contralateral minus ipsilateral activity) for valid cue sessions (red lines) and invalid cues (blue lines). Violin plots depict the PD amplitude (248-316 ms) in the two sessions, with the dots representing each subject. The solid and dotted lines indicate medians and quartiles, respectively. *p < 0.05. Con: contralateral to distractor cue; Ips: ipsilateral to distractor cue.

Thank you for reminding us of potential ERP activity before P_D_. According to Reviewer #3’s comments, more negative-going difference waves in the 220-250 ms period were defined as distractor-elicited N2pc. Further statistical analysis of N2pc showed that there was no significant difference before the P_D_ component between valid sessions and invalid sessions. As significant distractor-related N2pc was found in both valid sessions and invalid sessions, we cannot simply assume that the lack of P_D_ in the valid cue condition was driven by distractor-elicited N2pc. Please see Point 11 of Reviewer #3 for alternative explanations.

According to Reviewer #2’ s suggestion, the correlation plots and results between the CTF slope and P_D_ have been deleted, as no significant relationship was found after correcting for multiple testing.

12. I was a bit surprised to read a paper on spatial attention cuing and alpha suppression that did not cite the in princeps study of Michael Worden, where this effect was first reported. Also, and this may be quite self-serving, the first paper to implicate alpha-band activity in attentional suppression of visual distracting inputs (albeit in an intersensory design) was Foxe et al., 1998, which is also not cited. Overall, the scholarship in the paper is not at the level one would expect and a large swathe of relevant literature has been ignored. I've listed just a few papers below that tackle this precise issue (non-exhaustive).

Schneider D, Herbst SK, Klatt LI, Wöstmann M. Target enhancement or distractor suppression? Functionally distinct alpha oscillations form the basis of attention. Eur J Neurosci. 2022 Jun;55(11-12):3256-3265.

Worden MS, Foxe JJ, Wang N, Simpson GV. Anticipatory biasing of visuospatial attention indexed by retinotopically specific alpha-band electroencephalography increases over occipital cortex. J Neurosci. 2000 Mar 15;20(6):RC63.

Foxe JJ, Simpson GV, Ahlfors SP. Parieto-occipital approximately 10 Hz activity reflects anticipatory state of visual attention mechanisms. Neuroreport. 1998 Dec 1;9(17):3929-33.

**Response:** We are grateful to Reviewer #2 for reminding us of the relevant articles. We carefully read these articles and found that they are indeed related to our present study. We have cited these important references in the new version of the manuscript:

“Using an intermodal task, Foxe et al. (1998) reported enhanced alpha power in cortical areas when visual stimuli were task-irrelevant, thus suggesting that alpha-band activity is associated with distractor suppression. In a working memory task, alpha power was found to increase in anticipation of distractor (Bonnefond et al., 2012). Applying a cued spatial attention task, Worden et al. (2000) found posterior alpha-band activity which was larger contralateral to the ignored compared to the attended visual field . Given the tight link between spatial attention and alpha power, spatial attention bias is usually measured by alpha-band power activity (Schneider et al., 2022).”[Please see Lines 3-8 of Page 4]

Besides, we found several other relevant articles and cited them in the new version of manuscript:

Snyder, A. C., Foxe, J. J. (2010). Anticipatory attentional suppression of visual features indexed by oscillatory alpha-band power increases: a high-density electrical mapping study. The Journal of Neuroscience, 17;30(11):4024-32.

Foxe, J. J., Snyder, A.C. (2011) The Role of Alpha-Band Brain Oscillations as a Sensory Suppression Mechanism during Selective Attention. Frontiers in Psychology, 5(2):154.

Van Diepen, R. M., Foxe J. J, Mazaheri A. (2019). The functional role of alpha-band activity in attentional processing: the current zeitgeist and future outlook. Current Directions in Psychological Science, 29:229-238.

We have cited these important references in the new version of the manuscript: “Alpha-band activity can be interpreted as a neural signature of distractor inhibition (Snyder & Foxe, 2010; Foxe et al., 2011; Van Diepen et al., 2019; van Zoest et al., 2021) ” [Please see Lines 13-14 of Page 4].

**Reviewer #3 [Clayton Hickey]:**

1. p.3 - '…recent research suggests that distractors can be suppressed proactively… by showing that eye movements are less likely to be deployed to a cued distractor…' In general, this existing work from van Zoest, Huber, Weaver, & Hickey, 2021, JoN, does not get sufficient treatment in the current manuscript. Here, on p.3, that paper is cited as an eye movement study. In fact, that paper introduced many of the techniques used in the current paper in the analysis of lateralized alpha and N2pc / Pd, and directly addressed many of the same questions. Van Zoest et al. 2021 needs more prominent treatment through this submitted manuscript, with the novelty of the current study explicitly identified in reference to the existing results from that earlier paper.

**Response:** We thank Reviewer #3 for reminding us that the existing work from the relevant paper did not receive sufficient treatment in our manuscript. We carefully read this article again and found that it is related to our present study in the analysis of lateralized alpha and N2pc / P_D_ in addition to the work on eye movement.

We have cited this important reference (van Zoest et al., 2021) and discussed its importance:

“Alpha-band activity can be interpreted as a neural signature of distractor inhibition (Snyder & Foxe, 2010; Foxe et al., 2011; van Diepen et al., 2019; van Zoest et al., 2021)......These findings were complemented by a recent study reporting an increase in alpha power contralateral to predicted distractors (van Zoest et al., 2021).”[Lines 13-14 of Page 4]

“Recent studies found a reduction of the P_D_ following a predictable distractor, reflecting a decrease in attentional selection and suppression (Heuer & Schubo, 2020; van Zoest et al., 2021; van Moorselaar et al., 2019, 2020)” [Please see Lines 15-17 of Page 5]

“We also analyzed the mean performance for Experiment 1. Our behavioral results were consistent with previous studies (Noonan et al., 2016; Wang & Theeuwes 2018; Heuer & Schubo, 2020) that found no difference in reaction time and accuracy between validly and invalidly cued conditions. Please see supplementary materials for details.” [Lines 12-15 of Page 8]

“As shown in Figure 4...... which is called early P_D_ in previous studies (Weaver et al., 2017; van Zoest et al., 2021).” [Lines 9-11 of Page 17]

“Given that early P_D_ is related to initial processing of stimuli and is assumed to represent some low-level sensory imbalance between the two hemispheres (Weaver et al., 2017; Wang et al., 2019; van Zoest et al., 2021).”[Line 6-8 of Page 26]

“In addition, distractors can be suppressed proactively by showing that eye movements are less likely to be deployed to a cued distractor (van Zoest et al., 2021).” [Lines 18-20 of Page 26]

2. p.4, line 16 - The first mention of the Pd should have a citation to the original report of that component, Hickey, Di Lollo, & McDonald, 2009, JoCN.

**Response:** Thank you for this suggestion and the reminder of the related article. We have cited this important reference (Hickey et al., 2009, JoCN) in the new version of the manuscript. [Please see Line 13 of Page 5]

3. p.4 'There is still a lack of evidence for spatial suppression' This comment immediately follows a reference to van Zoest et al., 2021, which explicitly provided evidence for spatial suppression.

**Response:** Thank you for pointing out the problem that we missed the evidence for spatial suppression in this paper. We have carefully read this important paper and cited it. We rewrote the sentence “Recent studies found a reduction of the P_D_ following a predictable distractor, reflecting a decrease in attentional suppression (Heuer & Schubo, 2020; van Zoest et al., 2021, van Moorselaar et al., 2019, 2020), while there is still a lack of direct evidence on the relationship between anticipatory alpha activity and subsequent distractor-elicited P_D_ for spatial suppression, albeit the trial-wise magnitude of the pre-target alpha power was linked to the other ERP (van Zoest et al., 2021)” [Please see Lines 15-20 of Page 5]

4. p.4 - Target / distractor proximity effects on attentional selection have been investigated in depth, and there is the need to cite some of this literature. Eg. Mounts, 2000, P&P; Hilimire, Mounts, Park, & Corballis, 2009, Psychophysiology; Hickey & Theeuwes, 2011, APP.

**Response:** We are grateful to Reviewer #3 for reminding us of the relevant articles. We carefully read these articles and found that they are related to our results about target/distractor proximity effects on attentional selection. We have cited these important references in the new version of the manuscript.

“The spatial proximity of the distractor plays an important role in determining how proactive suppression impacts behavior (Caputo et al., 1998; Mounts, 2000; Hilimire et al., 2009). As the distance between the target and distractor increased, the accuracy of target selection improves monotonically while reaction time decreases monotonically (Caputo et al., 1998; Theeuwes et al., 2004; Hickey & Theeuwes, 2011). In recent decades, the dependency of performance on the distance between the target and distractor is calculated by the performance as a function of target-distractor distance (Koch et al., 2013; Gaspar et al., 2014; Wang & Theeuwes, 2018).” [Please see Lines 18-21 of Page 3, lines 1-2 of Page 4 in the new version of manuscript]

5. Two patterns emerge in analysis of proximity effects in the current data. Performance is better when distractors appear far from targets. But the cue is more effective when it identifies distractors that appear far from the target. This is confusing. Why would the cue benefit performance when it is least needed? This pattern needs more treatment in the paper; the paper doesn't currently give a clear account of what is happening here.

**Response:** We are grateful to Reviewer #3 for reminding us of the omission of an important discussion about behavioral results. As shown in Fig. L2, a significant relationship between cueing effects on alpha activity and ES slope was found in Experiment 1 (see Point 1 of Reviewer #1 for details). We discussed that “With respect to the invalid session, the channel response relatively increased as alpha power decreased at electrodes contralateral to the cued distractor location, where the target was enhanced more easily. Thus, we observed that the cue is more effective when it predicts distractors appearing further away from the target. In contrast, the channel response relatively decreased as alpha power increased at electrodes ipsilateral to the cued distractor location, where the target was suppressed more easily. Thus, the cue is less effective when it predict distractors appearing closer to the target.” We have added the above paragraph to the Discussion [Please see Lines 21-23 of Page 21, lines 1-4 of Page 22].

6. p.7 - 'Taken together, our preliminary results showed novel spatial behavioral changes…' The results appear to follow from Awh et al., 2003, JEPHPP; Munneke et al., 2008, Acta Psychologica; Chao, 2010, JEPHPP; van Zoest et al., 2021, JoN; Heuer & Schubo, 2020, Psychophysiology.

**Response:** We are grateful to Reviewer #3 for reminding us of the related articles. We carefully read these articles and found that they are related to our present study. We have cited these important references in the new version of the manuscript. We rewrote the sentence “As expected, these results are consistent with previous studies (Munneke et al., 2008; Chao, 2010; Heuer & Schubo, 2020; Feldmann-Wüstefeld et al., 2021; Awh et al., 2003) and provide clear evidence for a spatial gradient of suppression surrounding salient distractors.” [Please see Lines 14-16 of Page 7].

7. p.8 - '…from 264 to 744 ms…' '…from 196 to 648 ms…' How are latency intervals identified? Exactly how are these stats being conducted? Is each timepoint being analyzed? Is there a correction for family-wise error? Generally there needs to be better description of the statistical inference being employed here; p.10 - '… a significant Pd (248 - 316 ms; permutation test: p < 0.050)…' This statistical analysis is unclear. What's being done here? How is FWE controlled?

**Response:** We are grateful to Reviewer #3 for reminding us that the description of the statistical analysis is unclear. All latencies were identified by using standard cluster-based nonparametric tests (Maris and Oostenveld, 2007). First, we estimated the t statistic across participants for a contrast of interest at each time point, then defined observed clusters of consecutive above-threshold time points, and calculated the cluster mass (by summing all t values in an above-threshold cluster). Next, we randomly shuffle condition labels within participants (sign-flip for contrasts against zero) and extract the largest cluster mass produced by chance. This permutation step is performed 10,000 times to estimate the null distribution. The probability of the observed group-level cluster against chance is then derived as the rank order of the observed cluster relative to the null distribution. We have added some details to the manuscript as follows.

“All latencies were identified using standard cluster-based nonparametric tests (Maris & Oostenveld, 2007; Noonan et al., 2016).” [Lines 1-2 of Page 33]

“Resulting in a slope significantly (cluster-based permutation test: p < 0.050, two-tailed) different from zero in valid cue sessions (from 264 to 744 ms) and invalid cue sessions (from 196 to 648 ms)” [Lines 10-12 of Page 10]

“…revealing that a significant positive difference (248−316 ms; cluster-based permutation test: p < 0.050) in the invalid cue sessions and a significant cueing effect on the difference waveform was apparent at P7/8 electrodes (258−302 ms; cluster-based permutation test: p < 0.050).” [Lines 21-20 of Page 12, lines 1-3 of Page 13]

8. p.10 - In E1, distractor cueing removed a potential target location, increasing the probability that the target would appear in the contralateral hemifield. This potentially confounds any lateral effect associated with suppression with lateral effects associated with deployment of attention to the contralateral field. This is addressed somewhat in E2, but the problem and limitation in E1 needs to be explicitly identified in discussion / results section for E1, and the fix in E2 also needs more explicit description in the results section.

**Response:** Thank you for this constructive suggestion. We have added a more explicit description in the results section as follows.

“On the other hand, distractor cueing explicitly removed a potential target location in Experiment 1, increasing the probability that the target would appear in the contralateral hemifield. This potentially confounds the lateral effect related to suppression with lateral effects associated with the deployment of attention to the contralateral field. Here, to control the occurrence of a subsequent target location, we pseudorandomized the location of the target circle by specifying a uniform spatial probability of 4.25% on each lateral location and 33% on each midline location (see Figure 4A; right panel). This manipulation of the target appearing more often on the vertical meridian allowed us to isolate the lateralized brain activity related to distractor anticipation, which relies on the fact that stimuli on the vertical meridian target do not elicit the lateralized activities (Woodman and Luck, 2003; Hickey et al., 2006).” [Please see lines 18-22 of Page 15, lines 1-5 of Page 15 in the new version of the manuscript]

9. p.5 - The reduction of Pd following a valid distractor cue was also demonstrated by Heuer & Schubo, 2020, Psychophysiology; van Moorselaar, Lampers, Cordesius, & Slagter, 2020, eLlife; van Zoest et al., 2021, JoN. This is in addition to the current citation to van Moorselaar & Slagter, 2019, JoN.

**Response:** We are grateful to Reviewer #3 for reminding us of the related articles. We carefully read these articles and found that they are related to our present study. We have cited these important references in addition to the current citation.

“Recent studies found a reduction of the P_D_ following a predictable distractor, reflecting a decrease in attentional suppression (Heuer & Schubo, 2020; van Zoest et al., 2021, van Moorselaar et al., 2019, 2020)” [Please see Lines 15-17 of Page 5 in the new version of the manuscript].

10. p.11 - The use of vertical stimuli to study attentional deployment expressed in lateral EEG activity is critical to Experiment 2, makes the experiment valid and a significant advancement on E1. But the manipulation is not clearly identified in text; it is described in a figure caption and in the detailed methods. When describing this manipulation, it can be cited in reference to the papers where the method was first employed: Woodman & Luck, 2003, JEPHPP; Hickey, McDonald, & Theeuwes, 2006, JOCN. For target

**Response:** Thank you for this constructive suggestion and the reminder of the related articles. To identify the manipulation of Experiment 2 more clearly, we have cited these important references and added the following sentences to the Results: “On the other hand, distractor cueing explicitly removed a potential target location in Experiment 1, increasing the probability that the target would appear in the contralateral hemifield. This potentially confounds the lateral effect related to suppression with lateral effects associated with the deployment of attention to the contralateral field. Here, to control the occurrence of a subsequent target location, we pseudorandomized the location of the target circle by specifying a uniform spatial probability of 4.25% on each lateral location and 33% on each midline location (see Figure 4A; right panel). This manipulation of the target appearing more often on the vertical meridian allowed us to isolate the lateralized brain activity related to distractor anticipation, which relies on the fact that stimuli on the vertical meridian target do not elicit lateralized activities (Woodman and Luck, 2003; Hickey et al., 2006)”. [Lines 18-22 of Page 15, lines 1-5 of Page 15 in the new version of the manuscript]

11. Fig 3a - The Pd is preceded by contralateral negativity (ie. N2pc). This emerges in the latency that you'd expect in this kind of paradigm (ie. 200 - 250 ms) and appears to differ between the valid and invalid conditions, with the distractor-elicited N2pc larger in the valid condition than in the invalid condition. This suggests that participants are attentionally selecting the cued location more when it has been cued. They are then suppressing it less, as evident in a smaller Pd. This motivates a very different interpretation of the results than is currently offered, namely that subjects are not strongly suppressing the cued distractor, but rather strongly attending to it. This would be consistent with some of the extant literature (eg. Wang & Theeuwes, 2018, APP; Cunningham & Egeth, 2016, PsycSci).

- Can the difference waves of the two conditions be generated and plotted on the same axis? This facilitates consideration of these effects, which are small in comparison to the bilateral morphology of the ERP.

- Can the difference in N2pc between these conditions be statistically assessed?

- This result needs analysis and interpretation in the MS.

**Response:** Thank you for reminding us that this phenomenon was ignored in Experiment 1. According to Reviewer #3’s constructive suggestion, the difference waves of the two conditions have been generated and plotted on the same axis. “As shown in Fig. L7, the P_D_ is preceded by significant contralateral negativity in the valid session (172−236 ms) and in invalid session (192−218 ms; cluster-based permutation test: p < 0.050, two-tailed), which is called the distractor-elicited N2pc in previous studies (Wang & Theeuwes, 2018; Cunningham & Egeth, 2016). No significant difference in distractor-elicited N2pc between valid and invalid sessions was found (p > 0.225).”

“In Experiment 1, our ERP results show the distractor-elicited N2pc at approximately 150 ms poststimulus in both valid and invalid sessions. This finding suggests that participants are first attending to the cued location before suppressing the cued distractor. It is well documented that distractor inhibition may reflect a “search-and-inhibit” mechanism, whereby knowledge of the distractors initially paradoxically increases the attentional bias toward them and then guides attention away (Wang & Theeuwes, 2018; Cunningham & Egeth, 2016). Previous studies suggest that once valid information about distractors was provided in advance, people could not immediately escape attraction by the distractor information regardless of task demands (Cunningham & Egeth, 2016; Tsal et al., 2006). An alternative explanation stems from a white-bear metaphor that distractor cueing is the visual equivalent of telling someone not to think of a white bear effect (Wegner et al., 1987). Cueing distractor information is almost paradoxical to instructing someone to prepare to ignore something. They might first try to make an effort to minimize interference from cueing distractor information (Moher & Egeth, 2012) and then actively inhibit the potential distractor location. [Please see lines 15-23 of Page 26, lines 1-4 of Page 26 in the new version of the manuscript].

12. p11 - 'We used between-subject correlation analysis to investigate the relationship between cue-induced alpha activity and… Pd…' Why between subject? This is sensitive to third-variable problems. For example, those subjects with thinner skulls / better electrode contacts / less muscle noise / more motivation may show more prominent alpha and more prominent Pd, generating the observed correlations without any underlying causative relationship between alpha and the Pd. There needs to be some interpretation and treatment of the potential confounds that could underly between-subject correlations. There is also the opportunity to discuss the within-subject correlations identified by van Zoest et al., 2021, JoN.

**Response:** We are grateful to Reviewer #3 for reminding us of the potential confusion about between-subject correlation analysis. We also rewrote the sentence “Between-subject correlation analysis is sensitive to a third variable, such as better electrode contacts, less muscle noise, or more motivation. Several studies have investigated the alpha power at within-subjects level. van Dijk et al. (2008) found that the trial-wise variance of pretarget lateral alpha power is related to visual discrimination ability. van Zoest et al. (2021) further found that trial-wise variance of pretarget alpha power is related to the distractor-elicited N2pc and early P_D_. Hence, we further calculated the average single-trial P_D_ for each quartile to confirm the relationship between the alpha MI and subsequent P_D_ amplitude.” [Lines 2-7 of Page 18 in the new version of the manuscript].

13. P11 - 'A significant correlation was found for the valid-cue sessions… but no significant correlation was found for the invalid-cue sessions…' Are the correlations significantly different from each other?

**Response:** Thank you for reminding us to further calculate the difference between the correlations. According to Reviewer 2’s suggestions, we deleted the correlation plots and results between the CTF slope and P_D_ in Experiment 1, as no significant relationship was found after correcting for multiple testing. We analyzed the data from Experiment 2 and found no significant differences between the correlations for different conditions.

14. Figure 4 - The Pd results seem to show a qualitative difference, rather than a difference as a function of cue validity. That is, the Pd emerges strongly when the cued location has reasonable probability of containing the subsequent target but does not when the cued location is very unlikely to contain the target. This suggests that participants only used the cue when it identified a location that had low probability of containing a target, and this is consistent with the pattern of pre-stimulus alpha. The interpretation is importantly distinct from that currently presented in the paper, and the alternative deserves some treatment.

**Response:** We are grateful to Reviewer #3 for reminding us of an alternative explanation. “Before the experiment, participants were informed of the cue validity (high, low, or null) and its corresponding spatial probability of the target and distractor in advance. In Experiment 2, our behavioral results showed a significant cueing effect on mean RT and ES, suggesting that participants used the cue to guide attention deployments in both high-validity conditions and low-validity conditions. “Note that the target had exactly the same spatial probability across the different distractor predictive validity conditions, and the location of the subsequent target was totally independent of the distractor location. Thus, participants have no incentive to use distractor cues to infer the probability of a location containing a target.” We have added the above discussion to the new version of manuscript. [Please see lines 6-9 of Page 15 in the new version of the manuscript]

15. Fig 4 c - There is an early Pd in these results, emerging at about 150 ms post-stimulus. The early Pd is a functional component that appears associated with distractor suppression (eg. Weaver, van Zoest, & Hickey, 2017, Neuroimage; van Zoest et al, 2021, JoN). Is this contrast of cue results versus no-cue results significant in this early Pd interval? Is this early Pd predicted by pre-stimulus alpha laterality?

**Response:** Thank you for reminding us that this phenomenon was ignored in Experiment 2. According to Reviewer #3’s constructive suggestions, “As shown in Figure L8, the P_D_ is preceded by significant contralateral positivity in both the high- (152−186 ms) and the low-predictive validity trial (150−198 ms; cluster-based permutation test: p < 0.050, two-tailed), which is called early P_D_ in previous studies (Weaver et al., 2017; van Zoest et al., 2021). No significant difference in early P_D_ between null and other predictive validity conditions was found (ps > 0.458).”

**Fig. L8** ERP results during the stimulus period from Experiment 1.The corresponding difference waves (contralateral minus ipsilateral activity) for high- (red line), low- (blue line), and null (black line) predictive validity.The red, blue and black dashed lines indicate a significant difference from zero, and the dashed line with two colors indicates clusters with a significant difference between the two conditions (p < 0.05). Con: contralateral to distractor cue; Ips: ipsilateral to distractor cue. * p < 0.05.

According to Reviewer #3’s suggestion, we performed correlation analysis to investigate whether early P_D_ can be predicted by alpha MI. No correlation was found in either the high- or low-predictive validity trial at either the between-subject level (ps > 0.345) or the within-subject level (ps > 0.164).

“In Experiment 2, we only observed the early P_D_ at approximately 150 ms poststimulus in both high- and low-predictive validity trials but not in null-predictive validity trials. Early P_D_ is related to the initial processing of stimuli, it is assumed to reflect low-level sensory imbalance between the two hemispheres (Weaver et al., 2017; Wang et al., 2019; van Zoest et al., 2021). For null-predictive validity trial, the cue have no information about the visual field where the distractor occurs. Therefore, it is not strange that no early P_D_ was observed in null predictive validity. Furthermore, we inferred that no distractor-elicited N2pc, instead of early P_D,_ was observed in Experiment 2, which might also be due to the lack of the specific location where the distractor would appear. Accordingly, the early P_D_ observed in Experiment 2 might reflect initial and large-scale suppression of the visual field (left visual field or right visual field) where the distractor occurs.”We have also added the above discussion to the manuscript.

Minor:

16. Figure 2B - In the post-stimulus panel, the time-axis is not sufficiently labeled. The panel should identify that the image starts at -400 ms. As currently presented, it looks like it starts at 1200 ms, the label which applies to the post-cue panel… it's unnecessarily confusing.

**Response:** Thank you for pointing out this problem. We agree that the time axis is not sufficiently labeled in the original manuscript. We have modified the Figures in the new version of the manuscript by adding the label “-400” to the x-scale. Please see Figure 2B in the new version of manuscript.

17. p.8 - '…suggest that CTFs are sensitive to the attended distractor location…' Is the suggestion here that subjects attended to the cued location? Or that they inhibited it?

**Response:** We are grateful to Reviewer #3 for reminding us that the statement of the formulation “attended distractor location” may be ambiguous. It was intended that subjects inhibited rather than attended to the cued location. We have replaced “attended distractor location” with “inhibited distractor location” in the new version of the manuscript. [Please see line 11 of Page 9, line 6 of Page 10, line 15 of Page 11 in the new version of the manuscript]

18. p.8 - '…rise in tension…' What does this mean?

**Response:** Thank you for pointing out this problem. We meant it was a steep rise, but our expression is confusing. We have replaced the expression “an initial rapid rise in tension” with “an initial steep rise”. [Please see line 10 of Page 10 in the new version of the manuscript]

19. p.9 - '…channel response relatively increased at electrodes far away from the cued distractor…' This is perhaps correct if the term 'virtual electrodes' is used (and introduced prior to this point in the MS). But as it stands it is confusing as the reader assumes discussion is of actual electrodes, not CTF functions.

**Response:** We are grateful to Reviewer #3 for reminding us that this formulation is confusing. We have replaced “…channel response relatively increased at electrodes far from the cued distractor location, and channel response relatively decreased at electrodes close to the cued distractor location.” with “…channel response relatively increased at channels contralateral to the cued distractor location, and channel response relatively decreased at channels ipsilateral to the cued distractor location.” [Lines 3-5 of Page 11 in the new version of the manuscript]

20. p.22 - 'The EEG were amplified with…' to 'The EEG were filtered at…'

**Response:** Thank you for pointing out this error. We have replaced “The EEG were amplified with” with “The EEG were filtered at”. [Please see line 7 of Page 28 in the new version of the manuscript]

21. p.22 - '…digital bandpass of 0.1-40 Hz (6dB/octave…' One octave rolloff means that the filter passband would transition from 0 dB attenuation at 40 Hz. to -6 dB at 80 Hz. With a FIR implementation, that would mean that substantive power at 50 or 60 Hz line noise would remain in the signal. The filter described here is not particularly good for the purpose.

**Response:** Thank you for pointing out this problem. We have checked the filter parameter again, and we used “cutoff frequency (-6 dB) 0.05 to 40.05 Hz” rather than “6 dB/octave”. We apologize for this incorrect description of the filter used. We replaced "6 dB/octave" with "cutoff frequency (-6 dB) 0.05 to 40.05 Hz ". [Please see Line 9 of Page 28 in the new version of the manuscript]

22. p.25 - What decoding algorithm was employed? LDA, SVM, neural networks?

**Response:** The decoding algorithm employed was linear discriminant analysis (LDA). We have added this information to the Methods section and cited related references: “The decoding algorithm employed was linear discriminant analysis (Pedregosa et al., 2011), which is used in previous study (van Moorselaar et al., 2020).” [Please see lines 11-12 of Page 32 in the new version of the manuscript]

23. p.26 - What the Pd of maximum amplitude at P7/P8? It tends to be largest at PO7/PO8.

**Response:** We have plotted the topographic distribution of P_D_, which seems to be the largest at the PO7/PO8 locations in the international 10/10 system. However, the EEG data in our experiments were recorded using a 32-electrode elastic cap, which did not include the PO7/PO8 electrode. Therefore, we selected the P7/P8 electrode (closest to PO7/PO8) with the largest amplitude of P_D_, which was consistent with previous studies (e.g., Rodríguez-Martínez et al., 2021). As we replied to Reviewer #2, future work should explore brain activity by higher density montages (for details, please see point 4 of Reviewer #2).

24. p. 27 - What was the threshold for exclusion of a participant, in terms of excessive EEG artifacts?

**Response:** Thank you for pointing out the missing information on the threshold for the exclusion of a participant. We have added the threshold for exclusion of the participant “rejected trials > 30%”. [Please see line 4 of Page 34 in the new version of the manuscript].

25. p. 27 - All participants were right handed? Were they selected to be right handed? It's odd to have no left-handed participants in a 100 person sample.

**Response:** When we recruited the participants, a total of 110 right-handed and 4 left-handed participants signed up for the experiment. However, since our experiments in this study required the subjects to press buttons with only one hand, 110 right-handed subjects were selected to participate in this study to rule out the possible effect of handedness on lateralized brain activity. The 4 left-handed participants participated in another similar study in our lab.

In addition to the above-mentioned changes, a number of additional changes and corrections were made in order to make the paper more readable and understandable. We hope that these modifications, together with the above discussion, will satisfy the concerns expressed by both you and the three Reviewers.

Thank you again for all of the helpful and constructive comments and suggestions. We look forward to hearing more from you.

Sincerely yours,

Prof. Yan Song

State Key Laboratory of Cognitive Neuroscience and Learning

Beijing Normal University

Beijing, 100875, China

Tel: 86-10-5880-4267

Fax: 86-10-5880-6154

Email: songyan@bnu.edu.cn

### Reference

Awh, E., Matsukura, M., & Serences, J. T. (2003). Top-down control over biased competition during covert spatial orienting. Journal of Experimental Psychology: Human Perception and Performance, 29(1), 52–63.

Beatty, P. J., Buzzell, G. A., Roberts, D. M., Voloshyna, Y., & McDonald, C. G. (2021). Subthreshold error corrections predict adaptive post-error compensations. Psychophysiology, 58(6), e13803.

Bonnefond, M., & Jensen, O. (2012). Alpha oscillations serve to protect working memory maintenance against anticipated distracters. Current Biology, 22(20):1969-74.

Caputo, G. & Guerra, S. (1998). Attentional selection by distractor suppression. Vision Research, 38, 669–689.

Cazzato, V., Walters, E. R., Urgesi, C.(2021). Associations of observer’s gender, Body Mass Index and internalization of societal beauty ideals to visual body processing. Psychological Research. 85: 3026–3039.

Chang, S., Cunningham, C. A., Egeth, H. E. (2018). The power of negative thinking: Paradoxical but effective ignoring of salient-but-irrelevant stimuli with a spatial cue. Visual Cognition. 27: 1–15.

Chao, H. F. (2010). Top-down attentional control for distractor locations: the benefit of precuing distractor locations on target localization and discrimination. Journal of Experimental Psychology: Human Perception and Performance, 36(2), 303–316.

Cohen, M. X., & Donner, T. H. (2013). Midfrontal conflict-related thetaband power reflects neural oscillations that predict behavior. Journal of Neurophysiology, 110(12), 2752–2763.

Cunningham, C. A., & Egeth, H. E. (2016). Taming the white bear: initial costs and eventual benefits of distractor inhibition. Psychological Science, 27(4), 476–485.

Feldmann-Wüstefeld, T., Weinberger, M., & Awh, E. (2021). Spatially guided distractor suppression during visual search. The Journal of Neuroscience, 3180–3191.

Fortier-Gauthier, U., Dell’Acqua, R., & Jolicœur, P. (2013). The “red-alert” effect in visual search: Evidence from human electrophysiology. Psychophysiology, 50: 671–679.

Foster, J. J., Bsales, E. M., Jaffe, R. J., & Awh, E. (2017a). Alpha-band activity reveals spontaneous representations of spatial position in visual working memory. Current Biology, 27(20), 3216–3223.e6.

Foster, J. J., Sutterer, D. W., Serences, J. T., Vogel, E. K., & Awh, E. (2017b). Alpha-band oscillations enable spatially and temporally resolved tracking of covert spatial attention. Psychological Science, 28(7), 929–941.

Foxe, J. J., Simpson, G. V., & Ahlfors, S. P. (1998). Parieto-occipital approximately 10 Hz activity reflects anticipatory state of visual attention mechanisms. Neuroreport, 9(17), 3929–3933.

Gaspar, J. M., & McDonald, J. J. (2014). Suppression of salient objects prevents distraction in visual search. The Journal of Neuroscience, 34(16), 5658–5666.

Geyer, T., Müller, H. J., & Krummenacher, J. (2008) Expectancies modulate attentional capture by salient color singletons. Visual Research, 48(11): 1315–1326.

Heuer, A., & Schubö, A. (2020). Cueing distraction: electrophysiological evidence for anticipatory active suppression of distractor location. Psychological Research, 84: 2111–2121.

Hickey, C., & Theeuwes, J. (2011). Context and competition in the capture of visual attention. Attention, Perception & Psychophysics, 73(7), 2053–2064.

Hickey, C., Lollo, V. D., & McDonald, J. J. (2009). Electrophysiological indices of target and distractor processing in visual search. Journal of Cognitive Neuroscience, 21: 760–775.

Hickey, C., McDonald, J. J., & Theeuwes, J. (2006). Electrophysiological evidence of the capture of visual attention. Journal of Cognitive Neuroscience, 18(4), 604–613.

Hilimire, M. R., Mounts, J. R. W., Parks, N. A., & Corballis, P. M. (2009). Competitive interaction degrades target selection: An ERP study. Psychophysiology. 46: 1080–1089.

Jacques, C., & Rossion, B. (2007). Early electrophysiological responses to multiple face orientations correlate with individual discrimination performance in humans. NeuroImage, 36(3), 863–876.

Jensen, O., & Mazaheri, A. (2010). Shaping functional architecture by oscillatory alpha activity: gating by inhibition. Frontiers in Human Neuroscience, 4, 186.

Kennett, S., Eimer, M., Spence, C., & Driver, J. (2001). Tactile-Visual Links in Exogenous Spatial Attention under Different Postures: Convergent Evidence from Psychophysics and ERPs. Journal of Cognitive Neuroscience, 13(4), 462–478.

Koch, A. I., Müller, H. J., & Zehetleitner, M. (2013). Distractors less salient than targets capture attention rather than producing non-spatial filtering costs. Acta Psychologica, 144, 61–72.

Langner, O., Becker, E. S., Rinck, M., & van Knippenberg, A. (2015). Socially anxious individuals discriminate better between angry and neutral faces, particularly when using low spatial frequency information. Journal of Behavior Therapy and Experimental Psychiatry, 46, 44–49.

Li, J., Lim, J., Chen, Y., Wong, K., Thakor, N., Bezerianos, A., & Sun, Y. (2016). Mid-Task Break Improves Global Integration of Functional Connectivity in Lower Alpha Band. Frontiers in Human Neuroscience, 10, 304.

Liu, B., Nobre, A. C., & Ede, F. van. (2022). Functional but not obligatory link between microsaccades and neural modulation by covert spatial attention. Nature Communication, 13, 3503.

Lukashova-Sanz, O., Wahl, S., Wallis, T. S. A., & Rifai, K. (2021). The impact of shape-based cue discriminability on attentional performance. Vision, 5(2), 18.

Maris, E., & Oostenveld, R. (2007). Nonparametric statistical testing of EEG- and MEG-data. Journal of Neuroscience Methods, 164(1), 177–190.

McDonald, J. J., & Ward, L. M. (1999). Spatial relevance determines facilitatory and inhibitory effects of auditory covert spatial orienting. Journal of Experimental Psychology: Human Perception and Performance, 25, 1234–1252.

Moher, J., & Egeth, H. E. (2012). The ignoring paradox: cueing distractor features leads first to selection, then to inhibition of to-be-ignored items. Attention, Perception & Psychophysics, 74(8), 1590–1605.

Mounts, J. R. W. (2000). Attentional capture by abrupt onsets and feature singletons produces inhibitory surrounds. Percept Psychophys. 62: 1485–1493.

Munneke, J., Van der Stigchel, S., & Theeuwes, J. (2008). Cueing the location of a distractor: an inhibitory mechanism of spatial attention? Acta Psychologica, 129(1), 101–107.

Noonan, M. P., Adamian, N., Pike, A., Printzlau, F., Crittenden, B. M., & Stokes, M. G. (2016). Distinct mechanisms for distractor suppression and target facilitation. The Journal of Neuroscience, 36(6), 1797–1807.

Noonan, M. P., Adamian, N., Pike, A., Printzlau, F., Crittenden, B. M., Stokes, M. G. (2016). Distinct Mechanisms for Distractor Suppression and Target Facilitation. The Journal of Neuroscience. 1797–807.

Ortega-Alvarez, G., Matheus-Chacin, C., Garcia-Crespo, A. & Ruiz-Arroyo, A. (2022). Evaluation of user response by using visual cues designed to direct the viewer’s attention to the main scene in an immersive environment. Multimedia Tools and Applications, 1–27.

Pedregosa, F., Varoquaux, G., Gramfort, A., Michel, V., Thirion, B., Grisel, O., & Dubourg, V. (2011). Scikit-learn: machine learning in Python. Journal of Machine Learning Research 12:2830.

Pomerleau, V. J., Fortier-Gauthier, U., Corriveau, I., Dell'Acqua, R., & Jolicœur, P. (2014). Colour-specific differences in attentional deployment for equiluminant pop-out colours: evidence from lateralised potentials. International Journal of Psychophysiology, 91(3), 194–205.

Popov, T., Gips, B., Kastner, S., & Jensen, O. (2019). Spatial specificity of alpha oscillations in the human visual system. Human Brain Mapping, 40(15), 4432–4440.

Salamone, P. C., Legaz, A., Sedeño, L., Moguilner, S., Fraile-Vazquez, M., Campo, C. G., et al. (2021). Interoception primes emotional processing: Multimodal evidence from neurodegeneration. The Journal of Neuroscience. 41: 4276–4292.

Samaha, J., Sprague, T. C., & Postle, B. R. (2016). Decoding and reconstructing the focus of spatial attention from the topography of alpha-band oscillations. Journal of Cognitive Neuroscience, 28(8), 1090–141097. 15.

Schneider, D., Herbst, S. K., Klatt, L. I., & Wöstmann, M. (2022). Target enhancement or distractor suppression? Functionally distinct alpha oscillations form the basis of attention. The European Journal of Neuroscience, 55(11-12), 3256–3265.

Statsenko, Y., Habuza, T., Gorkom, K. N.-V., Zaki, N. & Almansoori, T. M. (2020). Applying the inverse efficiency score to visual–motor task for studying speed-accuracy performance while aging. Frontiers in aging neuroscience, 12, 574401.

Theeuwes, J., Kramer, A. F., & Kingstone, A. (2004). Attentional capture modulates perceptual sensitivity. Psychonomic Bulletin & Review, 11, 551–554.

Townsend, J. T., & Ashby, F. G. (1978). Methods of modeling capacity in simple processing systems. In N. J. Castellan & F. Restle (Eds.), Cognitive theory, vol. 3 (pp. 199–239). Hills-dale, NJ: Erlbaum.

Townsend, J. T., & Ashby, F. G. (1983).The stockastic modeling of elementary psychological processes. Cambridge: Cambridge University Press.

Tsal, Y., & Makovski, T. (2006). The attentional white bear phenomenon: the mandatory allocation of attention to expected distractor locations. Journal of Experimental Psychology. Human Perception and Performance, 32(2), 351–363.

van Diepen, R. M., Foxe J. J, Mazaheri A. (2019). The functional role of alpha-band activity in attentional processing: the current zeitgeist and future outlook. Current Directions in Psychological Science. 29:229-238.

van Dijk, H., Schoffelen, J. M., Oostenveld, R., & Jensen, O. (2008). Prestimulus oscillatory activity in the alpha band predicts visual discrimination ability. The Journal of Neuroscience, 28(8), 1816–1823.

van Driel, J., Gunseli, E., Meeter, M., & Olivers, C. N. L., (2017). Local and interregional alpha EEG dynamics dissociate between memory for search and memory for recognition. Neuroimage, 149, 114–128.

van Moorselaar, D., & Slagter, H. A. (2019). Learning what is irrelevant or relevant: expectations facilitate distractor inhibition and target facilitation through distinct neural mechanisms. The Journal of Neuroscience, 39(35), 6953–6967.

van Moorselaar, D., Lampers, E., Cordesius, E., & Slagter, H. A. (2020). Neural mechanisms underlying expectation-dependent inhibition of distracting information. Elife, 9: e61048.

van Zoest, W., Huber-Huber, C., Weaver, M. D., & Hickey, C. (2021). Strategic distractor suppression improves selective control in human vision. The Journal of Neuroscience, 7120–7135.

Wang, B., & Theeuwes, J. (2018). How to inhibit a distractor location? Statistical learning versus active, top-down suppression. Attention, Perception & Psychophysics, 80(4), 860–870.

Wang, B., van Driel, J., Ort, E., & Theeuwes, J. (2019). Anticipatory distractor suppression elicited by statistical regularities in visual search. Journal of Cognitive Neuroscience, 31(10), 1535–1548.

Weaver, M. D., Zoest, W. van & Hickey, C. (2017). A temporal dependency account of attentional inhibition in oculomotor control. Neuroimage, 147, 880–894.

Wegner, D. M., Schneider, D. J., Carter, S. R., & White, T. L. (1987). Paradoxical effects of thought suppression. Journal of Personality and Social Psychology, 53(1), 5–13.

Woodman, G. F., & Luck, S. J. (2003). Serial deployment of attention during visual search. Journal of Experimental Psychology: Human Perception and Performance, 29(1), 121–138.

Worden, M. S., Foxe, J. J., Wang, N., & Simpson, G. V. (2000). Anticipatory biasing of visuospatial attention indexed by retinotopically specific alpha-band electroencephalography increases over occipital cortex. The Journal of Neuroscience, 20(6):RC63.

Wöstmann, M., Störmer, V. S., Obleser. J., Addleman., Andersen. S. K., Gaspelin. N., et al. (2022). Ten simple rules to study distractor suppression. Progress in Neurobiology. 213: 102269.

Yeung, N., Bogacz, R., Holroyd, C.B., Nieuwenhuis, S., & Cohen, J. D. (2007). Theta phase resetting and the error-related negativity. Psychophysiology 44, 39–49.
