# Supplementary material for: Suppression of distracting inputs by visual-spatial cues is driven by anticipatory alpha activity
Source: PLoS Biol. 2023 Mar 8;21(3):e3002014. doi: 10.1371/journal.pbio.3002014 (PMC10027229; doi:10.1371/journal.pbio.3002014)
Supplement: Attachment — Submitted filename: Response_letter R2-6.docx [file pbio.3002014.s014.docx]

Please address reply to:

YAN SONG

STATE Key Laboratory OF COGNITIVE Neuroscience and learning

Beijing normal university

BEIJING,100875

PEOPLE’S REPUBLIC OF China

Telephone: (8610) 5880-4267

Fax: (8610) 5880-6154

Email:songyan@bnu.edu.cn

PLoS Biology Jan 26, 2023

**Ms. No.PBIOLOGY-D-22-01312_R2**

Title: Suppression of distracting inputs by visual-spatial cues is driven by anticipatory alpha activity

Corresponding Author: Prof. Yan Song

Authors: Chenguang Zhao, Yuanjun Kong, Dongwei Li, Jing Huang, Lujiao Kong, Xiaoli Li, Ole Jensen, Yan Song

**Dear Editor and Reviewer**,

We greatly appreciate the constructive comments from you and Reviewer. Since we received your comments, we have made a serious revision of our manuscript. 1) According to Editor’s constructive suggestions, we have modified the title to “Suppression of distracting inputs by visual-spatial cues is driven by anticipatory alpha activity”. 2) We have added and modified corresponding citations and rewritten some expressions to address Prof. Clayton Hickey's concerns. Our responses to the comments, as well as the modifications of the manuscript, are listed below:

**Point-by-point changes**

1. p.21 '…the cue is more effective when it predicts distractors further away from the target.' This is at odds with what we found in van Zoest et al. 2021. There, the cue was effective at reducing misdirection of the eyes to the distractor only when the target and distractor were in close spatial proximity (fig 2). There might be the opportunity to mention this disparity, note that the difference might result from the large difference in raw latency of eye movements versus manual response.

**Response:** Thank you for reminding us of the related results in this important reference. We have added the following sentences in the Discussion: “A recent study (van Zoest et al. 2021) reported that the cue was effective at reducing misdirection of the eyes to the distractor only when the target and distractor were in close spatial proximity, which seemed to be contrary to our behavioral results. We suggested that these inconsistent results might be explained by the large difference in raw latency of eye movements versus manual response.” [Please see lines 6-10 of Page 22 in the new version of the manuscript]

2. p.21 - '…by demonstrating that the spatial proximity of the distractor [effect] was influenced not only by statistical learning but also by the spatial cues.' This was also demonstrated in van Zoest et al. 2021, though the effect there was in the opposite direction.

**Response:** Thank you for the reminder, we have rewritten these sentences: “Previous studies have suggested that distractor spatial proximity effect was influenced not only by statistical learning (Wang & Theeuwes, 2018) but also by the spatial cues (van Zoest et al. 2021). Our results further extend our current understanding of the distractor spatial proximity effect on behavioral performance, and emphasize the importance of anticipatory alpha activities for suppression of distracting inputs.” [Please see lines 11-15 of Page 22 in the new version of the manuscript]

3. P26 - 'Given that the early Pd is related to… it is assumed to reflect low-level sensory imbalance…' That's not entirely accurate. There can be sensory imbalance in the latency of the early Pd, but the component can clearly have functional significance eg. Sawaki & Luck, 2010 APP; Weaver, van Zoest, & Hickey, 2017 Neuroimage. This line cites van Zoest et al., 2021, where the early Pd is shown to have functional significance, so the statement conflicts with the citation.

**Response:** We appreciate you for reminding us that this description is not entirely accurate. We have rewritten these sentences and cited the related articles: “Some studies proposed that early P_D_ is related to the initial processing of stimuli, which reflects low-level sensory imbalance between the two hemispheres (Fortier-Gauthier, 2013; Wang et al. 2019), and this component could also reflect functional distractor suppression during the early stage (van Zoest et al. 2021; Weaver, van Zoest, & Hickey, 2017; Sawaki & Luck, 2010).” [Please see lines 15-18 of Page 26 in the new version of the manuscript]

4. p.5 23 - 'We hypothesized that cueing the distractor location would influence on the spatial proximity of the distractor.' The effect is not on the spatial proximity of the distractor, but rather on how the spatial proximity of the distractor impacts another measure like behaviour or alpha. Strictly read, this line tells the reader that the cue changes the distance between target and distractor, which is not the intent. Also bottom p 7, top p.9, p.11 10, p21 17, elsewhere in MS.

**Response:** Thank you for reminding us that the description of “the spatial proximity of the distractor” may be ambiguous, and what we mean is indeed the effect on how the spatial proximity of the distractor impacts another measure like behaviour or alpha. According to your constructive suggestion, we modified the expressions in these parts of the new version of the manuscript as follows:

We have replaced “We hypothesized that cueing the distractor location would influence on the spatial proximity of the distractor.” with “We hypothesized that cueing the distractor location would influence the distractor spatial proximity effect on behavioral performance and alpha activities.”[Please see lines 1-3 of Page 6 in the new version of the manuscript]

We have replaced “We suggest that cueing distractor explicitly would influence the spatial proximity of the distractor.” with “We suggest that cueing distractor explicitly would influence the distractor spatial proximity effect on behavioral performance.”[Please see line 22 of Page 7, line 1 of Page 8 in the new version of the manuscript]

We have replaced “These results repeated the findings in Experiment 1, suggesting that the cueing distractor explicitly truly influenced the spatial proximity of the distractor, but not in relation to the specific color.” with “These results repeated the findings in Experiment 1, suggesting that the cueing distractor explicitly truly influenced the distractor spatial proximity effect on behavioral performance, but not in relation to the specific color. ”[Please see lines 3-5 of Page 9 in the new version of the manuscript]

We have replaced “We suggested that the spatial distribution of the alpha power response for an upcoming distractor can influence the spatial proximity of the distractor.” with “We suggested that the spatial distribution of the alpha power response for an upcoming distractor can influence the distractor spatial proximity effect on behavioral performance.”[Please see lines 10-12 of Page 11 in the new version of the manuscript]

We have replaced “This finding suggests that cueing distractors with variable predictive validity influences on the subsequent spatial proximity of the distractor. ” with “This finding suggests that cueing distractors with variable predictive validity influences on the subsequent distractor spatial proximity effect on behavioral performance.”[Please see lines 17-19 of Page 15 in the new version of the manuscript]

We have replaced “Behaviorally, we found changes of the spatial proximity of the distractor in Experiment 1” with “Behaviorally, we found changes of the cueing effect with different spatial proximity of the distractor in Experiment 1”[Please see lines 15-16 of Page 21 in the new version of the manuscript]

We have replaced “...... that the spatial distribution of alpha power response for upcoming distractors can influence the spatial proximity of the distractor. ” with “...... that the spatial distribution of alpha power response for upcoming distractors can influence the distractor spatial proximity effect on behavioral performance.”[Please see lines 21-23 of Page 21 in the new version of the manuscript]

We have replaced “In summary, cueing the distractor location by spatial cues in different circumstances could influence on the spatial proximity of the distractor.” with “In summary, cueing the distractor location by spatial cues in different circumstances could influence the distractor spatial proximity effect on behavioral performance and alpha activities.”[Please see lines 13-15 of Page 27]

5. P 4 9 - '…several recent studies have focused on the investigation of alpha power in terms of its hemispheric lateralization and spatial selectivity.'

**Response:** Thank you for your suggestion. We have replaced “...several recent studies have focused investigating alpha power in terms hemispheric lateralization and spatial selectivity” with “…several recent studies have focused on the investigation of alpha power in terms of its hemispheric lateralization and spatial selectivity.”[Please see lines 11-12 of Page 4 in the new version of the manuscript]

6. P 5 9 - '…albeit the trial-wise magnitude of the pre-target alpha power has been linked to lateral indices of attentional selection in the ERP [30].'

**Response:** Thank you for your suggestion. We have replaced “...albeit the trial-wise magnitude of the pre-target alpha power was linked to the other ERP [30]” with “…albeit the trial-wise magnitude of the pre-target alpha power has been linked to lateral indices of attentional selection in the ERP [30].”[Please see lines 19-20 of Page 5 in the new version of the manuscript]

7. There's room for more description of the cluster statistics. In particular, what was the cluster- defining statistical threshold?

**Response:** Thank you for your suggestion. We have rewritten the sentence and added the related statistical threshold: “All latencies were identified using standard cluster-based nonparametric tests with a cluster-defining threshold of p < 0.05.” [Please see lines 9-10 of Page 33 in the new version of the manuscript]

8. p.13 9 - Citation here to Cunningham & Egeth is wrong, there is no reference to N2pc in that paper.

**Response:** Thank you for pointing out this error. We have deleted this citation in the new version of the manuscript*.*[Please see line 14 of Page 13 in the new version of the manuscript]

We hope that these modifications, together with the above discussion, will satisfy the concerns expressed by both you and Prof. Clayton Hickey.

Thank you again for all of the helpful and constructive comments and suggestions. We look forward to hearing more from you.

**Sincerely yours**,

Prof. Yan Song

State Key Laboratory of Cognitive Neuroscience and Learning

Beijing Normal University

Beijing, 100875, China

Tel: 86-10-5880-4267

Fax: 86-10-5880-6154

Email: [songyan@bnu.edu.cn](mailto:songyan@bnu.edu.cn)

**References**

Fortier-Gauthier, U., Dell'acqua, R., & Jolicoeur, P. (2013). The “red-alert” effect in visual search: evidence from human electrophysiology. Psychophysiology, 50(7), 671–679.

Sawaki, R., & Luck, S. J. (2010). Capture versus suppression of attention by salient singletons: electrophysiological evidence for an automatic attend-to-me signal. Attention, perception & psychophysics, 72(6), 1455–1470.

van Zoest, W., Huber-Huber, C., Weaver, M. D., & Hickey, C. (2021). Strategic distractor suppression improves selective control in human vision. The Journal of Neuroscience, 41(33), 7120–7135.

Wang, B., & Theeuwes, J. (2018). How to inhibit a distractor location? Statistical learning versus active, top-down suppression. Attention, Perception & Psychophysics, 80(4), 860–870.

Wang, B., van Driel, J., Ort, E., & Theeuwes, J. (2019). Anticipatory distractor suppression elicited by statistical regularities in visual search. Journal of Cognitive Neuroscience, 31(10), 1535–1548.

Weaver, M. D., van Zoest, W. & Hickey, C. (2017). A temporal dependency account of attentional inhibition in oculomotor control. Neuroimage, 147, 880–894.
